# Unveiling the role of osteosarcoma-derived secretome in premetastatic lung remodelling

**DOI:** 10.1186/s13046-023-02886-9

**Published:** 2023-11-30

**Authors:** Sara F.F. Almeida, Liliana Santos, Gabriela Sampaio-Ribeiro, Hugo R.S. Ferreira, Nuno Lima, Rui Caetano, Mónica Abreu, Mónica Zuzarte, Ana Sofia Ribeiro, Artur Paiva, Tânia Martins-Marques, Paulo Teixeira, Rui Almeida, José Manuel Casanova, Henrique Girão, Antero J. Abrunhosa, Célia M. Gomes

**Affiliations:** 1 Institute for Nuclear Sciences Applied to Health (ICNAS) and Coimbra Institute for Biomedical Imaging and Translational Research (CIBIT), University of Coimbra, Coimbra, 3000-548 Portugal; 2 Faculty of Medicine, Coimbra Institute for Clinical and Biomedical Research (iCBR), University of Coimbra, Coimbra, 3000-548 Portugal; 3 Center for Innovative Biomedicine and Biotechnology Consortium (CIBB), University of Coimbra, Coimbra, 3000-548 Portugal; 4 Clinical Academic Center of Coimbra (CACC), Coimbra, 3000-075 Portugal; 5 Pathology Department, Centro Hospitalar e Universitário de Coimbra, Coimbra, 3004-561 Portugal; 6 Multidisciplinary Institute of Ageing (MIA), University of Coimbra, Coimbra, Portugal; 7 Instituto de Investigação e Inovação em Saúde (i3S), Porto, 4200-135 Portugal; 8 Flow Cytometry Unit, Department of Clinical Pathology, Centro Hospitalar e Universitário de Coimbra, Coimbra, Portugal; 9 Tumor Unit of the Locomotor Apparatus (UTAL), Orthopedics Service, Coimbra Hospital and University Center (CHUC), University Clinic of Orthopedics, Coimbra, 3000-075 Portugal

**Keywords:** Osteosarcoma, Lung Metastasis, Premetastatic niche, Extracellular matrix, Neutrophils, Fibroblasts, EFEMP1

## Abstract

**Background:**

Lung metastasis is the most adverse clinical factor and remains the leading cause of osteosarcoma-related death. Deciphering the mechanisms driving metastatic spread is crucial for finding open therapeutic windows for successful organ-specific interventions that may halt or prevent lung metastasis.

**Methods:**

We employed a mouse premetastatic lung-based multi-omics integrative approach combined with clinical features to uncover the specific changes that precede lung metastasis formation and identify novel molecular targets and biomarker of clinical utility that enable the design of novel therapeutic strategies.

**Results:**

We found that osteosarcoma-bearing mice or those preconditioned with the osteosarcoma cell secretome harbour profound lung structural alterations with airway damage, inflammation, neutrophil infiltration, and extracellular matrix remodelling with increased deposition of fibronectin and collagens by resident stromal activated fibroblasts, favouring the adhesion of disseminated tumour cells. Systemic-induced microenvironmental changes, supported by transcriptomic and histological data, promoted and accelerated lung metastasis formation. Comparative proteome profiling of the cell secretome and mouse plasma identified a large number of proteins involved in extracellular-matrix organization, cell-matrix adhesion, neutrophil degranulation, and cytokine-mediated signalling, consistent with the observed lung microenvironmental changes. Moreover, we identified EFEMP1, an extracellular matrix glycoprotein exclusively secreted by metastatic cells, in the plasma of mice bearing a primary tumour and in biopsy specimens from osteosarcoma patients with poorer overall survival. Depletion of EFEMP1 from the secretome prevents the formation of lung metastasis.

**Conclusions:**

Integration of our data uncovers neutrophil infiltration and the functional contribution of stromal-activated fibroblasts in ECM remodelling for tumour cell attachment as early pro-metastatic events, which may hold therapeutic potential in preventing or slowing the metastatic spread. Moreover, we identified EFEMP1, a secreted glycoprotein, as a metastatic driver and a potential candidate prognostic biomarker for lung metastasis in osteosarcoma patients.

**Graphical abstract:**

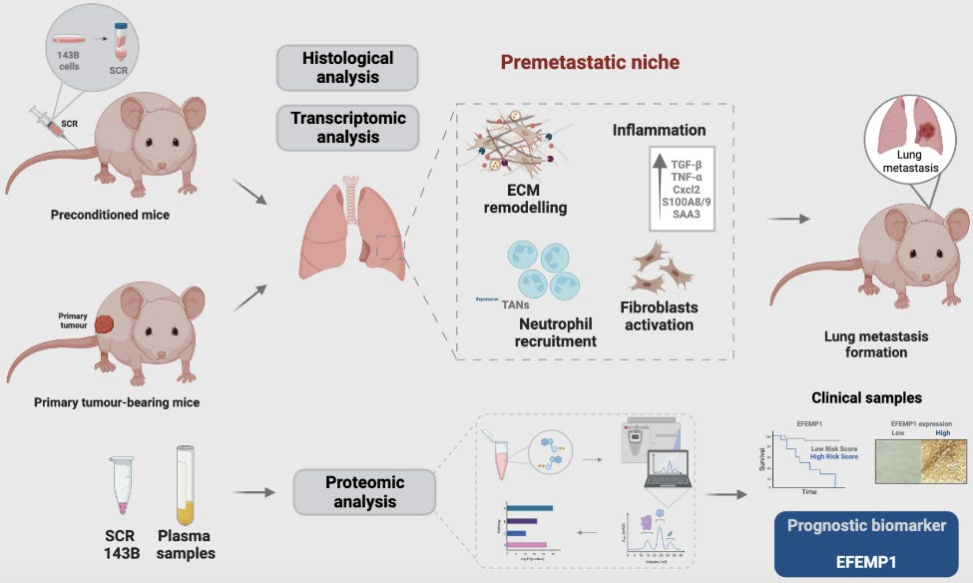

Osteosarcoma-derived secreted factors systemically reprogrammed the lung microenvironment and fostered a growth-permissive niche for incoming disseminated cells to survive and outgrow into overt metastasis.

- Daily administration of osteosarcoma cell secretome mimics the systemic release of tumour-secreted factors of a growing tumour in mice during PMN formation;
- Transcriptomic and histological analysis of premetastatic lungs revealed inflammatory-induced stromal fibroblast activation, neutrophil infiltration, and ECM remodelling as early onset pro-metastatic events;
- Proteome profiling identified EFEMP1, an extracellular secreted glycoprotein, as a potential predictive biomarker for lung metastasis and poor prognosis in osteosarcoma patients.
- Osteosarcoma patients with EFEMP1 expressing biopsies have a poorer overall survival.

**Supplementary Information:**

The online version contains supplementary material available at 10.1186/s13046-023-02886-9.

## Background

Osteosarcoma (OS) is the most prevalent primary malignant bone tumour that primarily affects children and adolescents [1, 2]. OS has a high propensity to metastasize to the lungs, accounting for 90% of metastatic sites, making it the major cause of morbidity and mortality [3]. The estimated 5-year survival rate of OS patients with localized disease is 70% but drops dramatically to less than 20% in patients who developed metastasis owing to the ineffectiveness of current therapeutic strategies and late diagnosis [4, 5].

It is speculated that over 80% of OS patients have undetectable lung micrometastases at initial diagnosis [6] that eventually progress to death despite receiving aggressive multiagent (neo)adjuvant chemotherapy. Despite intensive efforts to improve the outcome of OS patients, the survival benefits reported in clinical trials evaluating new therapies are quite marginal because of metastatic disease [7]. Mechanistically, the development of distant metastasis is a complex and multi-step process that is distinct from primary tumour formation. The dynamic crosstalk between cancer cells and the local microenvironment is recognized as a critical regulator of tumour progression and metastasis [8–10]. It is now evident that primary tumours prepare in advance and remotely a supportive and receptive microenvironment in specific secondary organs, the so-called premetastatic niche (PMN), for forthcoming tumour cells to adapt and survive [11, 12]. PMN formation encompasses a series of sequential and dynamic events in premetastatic organs primed by tumour-secreted factors and extracellular vesicles (EVs). This process involves a complex interplay of these factors with local stromal cells and tumour-mobilised bone marrow-derived cells (BMDCs) [13, 14]. Vascular leakage, abnormal extracellular matrix (ECM) remodelling, and immunosuppression have been identified as pro-metastatic events [12, 13]. Several studies have already demonstrated these alterations in different tumour types, including melanoma, breast, and colorectal cancers [15–17].

Therapeutic strategies targeting PMN formation at organ-specific sites offer an opportunity to prevent or suppress metastasis formation and are currently a hot topic in cancer research [18, 19]. The identification of potential druggable targets requires a tumour-specific understanding of the cellular and molecular mechanisms involved in establishing organ-specific PMNs. Despite advances in this topic, sarcomas have been less studied, and the mechanisms underlying the development of organotropic lung metastasis are not clearly understood [5, 20].

The cancer secretome is a reservoir of potential biomarkers and signalling biomolecules relevant to tumour progression and metastasis [21, 22]. This class of proteins has been studied in the search for cancer biomarkers and to understand their mechanistic role in tumour progression [23, 24]. Jerez and colleagues [25] conducted a proteomic analysis of the secretome of human OS cells and identified specific proteins in both exosomes and soluble fractions, involved in biological functions (angiogenesis, cellular adhesion, and migration) related to tumour progression and metastasis. Recently, Mazumdar et al. [26] explored the functional role of OS-derived EVs in driving lung metastatic colonization. Despite having observed preferential accumulation of EVs in the lungs, the induced stromal changes did not result in an increased tumour burden, suggesting that EVs dictate organotropism but additional tumour-secreted factors are required to establish a functional PMN [26].

In this study, we evaluated how primary OS systemically reprograms the lung microenvironment to establish a permissive pro-metastatic niche for subsequent metastasis formation using two murine models and a multi-omics approach. Transcriptomic data and tissue analysis uncovered ECM remodelling with deposition of cell adhesion-promoting proteins by stromal-activated fibroblasts, and neutrophil recruitment as early pro-metastatic events guiding lung metastasis. Furthermore, we identified EFEMP1, a glycoprotein secreted by tumour cells, as a key player in the development of lung metastasis and a potential prognostic biomarker in OS patients.

## Methods

### Cell culture and Infection with a lentivirus encoding luciferase

The human OS cell line 143B was purchased from the American Type Culture Collection (ATCC, Manassas, USA) and cultured in Eagle’s minimum essential medium (EMEM, Sigma Aldrich, UK), supplemented with 10% heat-inactivated fetal bovine serum (FBS, Gibco, Paisley, UK), 0.015 mg/ml 5-bromo-2’-deoxyuridine (BrdU, Sigma Aldrich, St. Louis, MO, USA), 1.0 mM sodium pyruvate (Gibco, Grand Island, USA), and 1% antibiotic-antimycotic (Gibco, Grand Island, USA). The cells were maintained under standard adherent conditions in a humidified incubator with 5% CO_2_ at 37 °C. Cells were authenticated by immunohistochemistry with antibodies against vimentin (Invitrogen, Thermo Fischer Scientific, Netherlands) and Ki-67 (clone MIB-1; Dako) and haematoxylin and eosin (H&E). The 143B cells were stably transduced with a lentivirus encoding Luciferase as described elsewhere [27].

For secretome (SCR) preparation, 143B cells (25 × 10^3^ cells/cm^2^) were cultured in an exosome-free culture medium for 24 h. The exosome-depleted medium was prepared by ultracentrifugation of 50% FBS in the appropriate cell culture medium (120 000 g, 16 h). The supernatant was collected after 24 h of incubation, centrifuged to remove cell debris and concentrated with Amicon Ultra-15 Centrifugal Filters (10 kDa molecular weight cut-off, Merck Millipore Ltd, Carrigtwohill, Ireland). Aliquots were stored at -80ºC until usage.

### EFEMP1 knockdown

Small interfering RNA (siRNA) knockdown of EFEMP1 in 143B cells was performed using the siGENOME human EFEMP1 siRNA SMARTpool (M-011855-01-0010). At 50% confluency cells were transfected with 20nM siRNA and a nontargeting (NT) control sequence (D-001810-10), both from Dharmacon Inc (Lafayette, USA), using Lipofectamine™ 3000 (Thermo Fisher Scientific, Waltham, USA) according to the manufacturer’s recommendations. After 48 h, the culture medium was replaced by an exosome-free medium, and the cells were maintained in culture for another 24 h afterwards the secretome was collected. The knockdown efficiency was evaluated by western blot and ELISA.

### Animal studies

Athymic Swiss nude (Foxn1^nu/nu^) mice, male or female, (8–12 weeks old, 20–30 g) were purchased from the Institute for Clinical and Biomedical Research (iCBR, Coimbra, Portugal) of the Faculty of Medicine of the University of Coimbra and housed under pathogen-free conditions in individually ventilated cages, with controlled temperature/humidity (22 °C/55%) environment on a 12 h light-dark cycle and with food and water ad libitum.

#### Premetastatic niche formation

Animals were randomized into two groups. A group was injected subcutaneously into the lower flank with 5 × 10^5^ of 143B-Luc^+^/100 µL PBS for primary tumour formation. The tumour growth was monitored every two days in two dimensions using a digital calliper, and mice were sacrificed when the tumour reached 50–60 mm^3^ in volume. Tumour volumes were calculated using the modified ellipsoid formula V = A × B^2^/3 (A length; B width).

The second group received a daily intraperitoneal (*i.p*.) injection of 25 µL of the 143B-derived SCR or the vehicle for 1 week, following which they were sacrificed. Animals were euthanized by cervical dislocation, and peripheral blood and lung tissue were harvested for further analysis. Peripheral blood samples were collected to K_3_EDTA tubes (Greiner bio-one, Kremsmunster, Austria) from anaesthetized animals via cardiac puncture before euthanasia. Plasma was separated by centrifugation at 3000 rpm for 10 min at 4ºC, and aliquots were stored at -80ºC until usage.

#### Mouse models of Lung Metastasis

For experimental lung metastasis formation, animals were injected intravenously (*i.v*.) into the lateral tail vein with 1.5 × 10^6^ of 143B-Luc^+^ cells/100 µL PBS. Another set of animals received daily intraperitoneal injections of 25 µL of the SCR for 1 week prior to the *i.v.* injection of 143B-Luc^+^ (1.5 × 10^6^ cells/100 µL PBS). A separate group received the SCR of EFEMP1-knockdown 143B cells, followed by tail vein injection of the 143B-Luc^+^ cells, as described above.

For spontaneous lung metastasis formation, animals were injected subcutaneously in the lower flank with 5 × 10^5^ of 143B-Luc^+^ cells/100 µL PBS. After reaching a maximum volume of 60–100 mm^3^, subcutaneous tumours were surgically excised and the skin incision was sutured with the animals under anaesthesia (10 mg/kg; Rompun 2%, Kiel, Germany).

Animals were monitored weekly for lung metastasis formation during a maximum of 60 days by bioluminescence imaging (BLI) on an IVIS Lumina XR (Caliper Life Sciences Inc., PerkinElmer, Massachusetts, USA). Images were acquired after *i.p.* injection of D-Luciferin (150 mg/kg), with the animals anaesthetized with 2.5% of isoflurane (Virbac, Carros, France) in 100% O_2_. Bioluminescent images were analysed using the Living Image software version 4.10 (Xenogen, Alameda, California). A region of interest was drawn around the lesions for the quantification of the bioluminescent signal. Values are expressed photons/sec/cm^2^/sr. At the end of the study, animals were euthanized by cervical dislocation and peripheral blood and metastatic lungs were collected for histopathological analysis.

### Clinical samples

Paraffin-embedded biopsy specimens were obtained retrospectively from 29 patients diagnosed with OS. At the time of diagnosis, all patients had strictly localized disease and were naïve to any chemotherapy. Of the 29 patients, 26 were classified as having conventional high-grade OS while the remaining 3 were classified as low-grade. Except for those with low-grade, all patients underwent chemotherapy prior to surgery, and all received adjuvant chemotherapy. The follow-up period ranged from 1 year to at least 10 years, or until death.

The specific characteristics of the patients are summarized in Table 1.

**Table 1:** Clinicopathological features of osteosarcoma patients

Number of patients	29
**Age**	
Mean (range)	33.6 (15–78)
**Variable**	**Number (%)**
**Gender**	
Male	15 (52)
Female	14 (48)
**Primary tumour location**	
Femur	16 (55)
Tibia	5 (17)
Humerus	2 (7)
Fibula	2 (7)
Others	4 (14)
**Tumour grade**	
High grade	26 (90)
Low grade	3 (10)
**Metastasis**	12 (41)
**Disease-free interval (DFI)**	**Months**
Mean (range)	41.4 (6-159)

#### Scanning electron microscopy (SEM)

Small fragments of resected lung tissues and decellularized matrices were fixed with 2% glutaraldehyde and examined in a scanning electron microscope (Flex SEM 1000, HITACHI), under variable pressure scanning using an accelerating voltage of 5–10 kV.

### Transmission electron microscopy (TEM)

Resected lung tissues were sectioned into small fragments (1 mm^3^) and fixed for 2 h in 2.5% glutaraldehyde buffered with 0.1 M cacodylate buffer (pH 7.4), followed by a post-fixation in 1% osmium tetroxide (OsO_4_) for 1.5 h. After washing, samples were incubated with 1% aqueous uranyl acetate for 1 h, for contrast enhancement. Samples were then dehydrated in a graded acetone series (30–100%) followed by resin embedding using an epoxy embedding kit (Fluka Analytical, Sigma Aldrich, Germany). Ultra-thin sections were obtained with a Leica EM UC6 (Leica Co; Austria) ultramicrotome, mounted on copper grids and stained with lead citrate 0.2% for 7 min. Images were acquired on a Tecnai G2 Spirit Bio Twin electron microscopy at 100 kV (FEI) and AnalySIS 3.2 software.

### Histopathological analysis and immunostaining

For murine models, lung tissues and primary tumours were fixed in 4% paraformaldehyde (PFA) and processed for paraffin embedding. Sections of 4 μm were stained with haematoxylin and eosin (H&E, Sigma-Aldrich, St. Louis, MO, EUA) or antibodies against fibronectin (ab2413, Abcam, USA), alpha-smooth muscle actin (α-SMA, ABT 1487, Millipore, Darmstadt, Germany) and vimentin (V9; Ventana, Arizona, EUA). Antigen retrieval was performed by immersing slides in EDTA-Tris buffer (pH 8) for 8 min at 95 ºC and then blocked with a buffered hyper protein solution for 4 min to avoid nonspecific bonds. Immunostaining was performed using a Ventana Marker Platform Benchmark Ultra IHC/ISH with the resource of a multimeric indirect free biotin detection system - Optiview DAB IHC Detection Kit (Ventana Medical Systems, Arizona, EUA), according to the manufacturer instructions. A Gordon’s and Sweet silver staining was performed for the detection of reticulin fibers. Slides were observed under a light microscope Nikon Eclipse 50 I and images were captured with a Nikon-Digital Sight DS-Fi1 camera. Architectural changes were evaluated, and inter-alveolar septal thickness was measured in randomly selected H&E-stained sections.

Clinical samples were formalin-fixed for 24 h and then decalcified with 10% nitric acid for 1–5 days based on tissue hardness, with daily integrity checks. The samples were paraffin-embedded and sectioned into 4 μm tissue slices for immunostaining with anti-EFEMP1/fibulin 3 monoclonal antibody (1:500, ab256457, Abcam). Hematoxylin-eosin staining was performed using standard methods as a counterstain. Immunohistochemistry positivity was considered when at least 1% of the viable neoplastic cells showed cytoplasmic and/or membrane expression of any intensity (weak, moderate or strong). The sections were assessed by an experienced pathologist.

### Primary lung fibroblasts isolation and culture

Lungs were harvested, washed in PBS, minced with scissors, and enzymatically digested with 0.1% collagenase A (Roche, Mannheim, Germany) and dispase II (2.4 U/mL; Gibco, Japan) for 90 min at 37 ºC, and then filtered through a 70 μm filter strainer and washed with a physiological saline solution containing 0.05 M EDTA. The obtained cell suspension was plated into 1% gelatin pre-coated dishes and maintained in RPMI 1640 (Sigma-Aldrich, UK) supplemented with 15% FBS and 1% antibiotic-antimycotic at 37 ºC under 5% CO_2_. All experiments were performed with early passages fibroblasts (P2-P4).

### Decellularization

#### Lung fragments

Small lung fragments (2 × 2 mm) were incubated in hypotonic buffer (10 mM Tris HCl/0.1% EDTA, pH 7.8) for 18 h at room temperature, and then washed in PBS (3x, 1 h) and immersed in a detergent solution (0.2% SDS/10 mM Tris HCl, pH 7.8) for 24 h at room temperature. Fragments were washed in hypotonic buffer (3x, 20 min) and incubated in DNAse solution (50 U/ml DNAse/ 10mMTris HCl, pH 7.8) for 3 h at 37 °C. Decellularized matrices were washed in PBS (2x, 20 min) to remove the residual detergent and DNAse solution and were maintained under sterile conditions at 4ºC in PBS until use. All steps were performed under agitation at 165 rpm.

#### Fibroblasts

Fibroblast cell sheets were incubated with buffer I (1 M NaCl, 5 mM EDTA, 10 mM Tris/HCl pH 7.4) for 1 h at room temperature, washed with PBS (3x, 10 min) and then exposed to buffer II (0.5% [w/v] SDS, 25 mM EDTA, 10 mM Tris/HCl pH 7.4) for 30 min with agitation.

### Immunofluorescence

Primary fibroblasts were fixed with 4% paraformaldehyde (PFA, Sigma-Aldrich, St. Louis, MO, USA) for 20 min, permeabilized with 0.2% TritonX-100 (Sigma-Aldrich, St. Louis, MO, USA) for 10 min, and blocked with 3% bovine serum albumin (BSA, Sigma-Aldrich, St. Louis, MO, USA) for 1 h at room temperature. Afterwards, the cells were incubated with the primary antibodies against α-SMA (1:200, ABT 1487; Millipore, Darmstadt, Germany), fibroblast activation protein (FAP) (1:150, PA5-99313, Thermo Fisher Scientific, USA), vimentin (1:200, SP20, Thermo Fisher Scientific, USA) and fibronectin (1:200, ab2413, Abcam, Cambridge, UK) overnight at 4 ºC. The fibroblasts were then incubated with secondary antibody Alexa Fluor 568 or 488 (1:200, Invitrogen, USA) for 1 h at room temperature in the dark, and nuclei were counter with 2 mg/mL Hoechst 33,342 (Sigma-Aldrich, USA).

For immunofluorescence staining with Alexa Fluor 555 phalloidin, cells were fixed with PBS containing 4% sucrose and 4% paraformaldehyde for 10 min, permeabilized with 1% TritonX-100 for 10 min and blocked with 1% BSA for 45 min at room temperature , and then incubated with Alexa Fluor 555 phalloidin (1:40, Abcam, Cambridge, MA, USA) for 30 min at RT. Nuclei were stained with 2 mg/mL Hoechst 33,342 (Sigma-Aldrich, Buchs, Switzerland). The chamber slides were mounted in the Vectashield Mounting Medium (Vectashield, Vector Laboratories, United States).

Decellularized lung fragments and fibroblast cell sheets were processed for immunofluorescence as described above and stained with the primary antibodies against fibronectin (1:200) and collagen IV (1:200 ab19808; Abcam, Cambridge, UK) and then incubated with secondary antibody Alexa Fluor 488 (1:200, Invitrogen, USA). Images were captured in Carl Zeiss Axio Observer Z1 inverted microscope (Carl Zeiss, Thornwood, NY) and processed using the ImageJ 1.52p software (National Institutes of Health, USA).

### Cell migration

Cell migration was analysed using the wound-healing assay. Confluent monolayers of fibroblasts were manually scratched with a 200 µL sterile pipette tip. Photographs were taken immediately after scratching (baseline) and at 6 and 24 h in a Carl Zeiss Axio Observer Z1 inverted microscope (Carl Zeiss, Thornwood, NY). The wound closure at each time-point was quantified using ImageJ 1.52p software (National Institutes of Health, USA) and normalized to the baseline.

### Lung transcriptomic analysis

Total RNA was extracted from lung tissues using TRIzol reagent (Ambion by Life Technologies, USA) according to the manufacturer’s instructions. RNA was eluted in 30–40 µL RNase-free water. The concentration and quality of extracted RNA were determined with a NanoDrop spectrophotometer (Thermo Scientific, Wilmington, USA). RNA was stored at 80 ºC until use. Single-end sequencing was performed using the library prep kit TruSeq and the sequencing kit NovaSeq6000 SP Flowcell 100 cycles (Illumina, Inc.) for Mus musculus (Ensembl.GRCm38.82). The transcriptomic sequencing of RNA was performed on the Illumina NovaSeq 6000 instrument, at the VIB Nucleomics Core (www.nucleomics.be). The reads pre-processing was performed by VIB Nucleomics Core and involved: quality trimming (FastX 0.0.14, HannonLab), adapter trimming (cutadapt 1.15) [28], quality filtering (FastX 0.0.14 and ShortRead 1.40.0) [29] and removal of contaminants (bowtie 2.3.3.1). The pre-processed reads were then aligned to the reference genome of Mus_musculus.Ensembl.GRCm.38.82 using STAR 2.5.2b [30] and SAMtools 1.5 [31] Bioconductor package. The expression levels of the read overlapping genes were computed using the EDASeq package [32] for the within-sample and between samples normalizations. The differentially expressed genes were estimated by fitting a negative binomial generalized linear model using the edgeR 3.24.3 package [33]. The resultant p-value was corrected for multiple tests with Benjamini-Hochberg to control the false discovery rate (FDR).

Unless stated otherwise, the following bioinformatics analyses were conducted using the R programming language (version 4.1.1) in the RStudio integrated development environment. The volcano plots were achieved using the ggplot2 [34] (version 3.3.5) and ggrepel (version 0.9.1) packages. The Venn diagrams were generated with the webtool available at bioinformatics.psb.ugent.be/webtools/Venn/. The clusterProfiler [35] version 4.0.5) package was used to perform the gene set enrichment analyses (GSEA) for the Gene Ontology (GO) terms and Kyoto Encyclopedia of Genes and Genomes (KEGG) pathways. Genes showing a p-value higher than 0.05 were excluded from the gene set. Heat maps were made with the ComplexHeatmap [36] (version 2.8.0) package. Protein-protein interaction (PPI) network of DEGs was visualized using the Search Tool for the Retrieval of Interacting Genes/Proteins (STRING) database (http://string-db.org/). Graphics were refined and figures were assembled in Adobe Illustrator 2019 (version 23.1.1).

### Flow cytometry

Fresh lung tissue was minced with fine scissors and subjected to mechanical dissociation (GentleMACS, Miltenyi Biotech, Germany). The tissue homogenates were filtered gently through a 40 µm cell strainer and centrifuged at 600 g for 15 min at RT. The isolated single-cell suspension and peripheral leukocytes were fluorescently stained with anti-mouse CD11b-FITC (1:200, BioLegend, 101205) and Ly6G-PE (1:80, BioLegend, 127607), CD68-APC (1:300, BioLegend, 137007), CD19-PerCP (1:200, BioLegend, 115531) and CD45-PB (1:200, BioLegend, 103125). FACS Lysing solution (BD Sciences) was added to the samples for lysing red blood cells. Data were collected on a FACS Canto^™^ II (BD Biosciences, USA) and analysed using Infinicyt V.1.8 software (Cytognos, Salamanca, Spain).

### RNA extraction and qRT-PCR

Total RNA from lung tissue was extracted using TRIzol reagent (Ambion by Life Technologies, USA) according to the manufacturer’s instructions. The concentration and quality of extracted RNA were determined using a NanoDrop spectrophotometer (Thermo Scientific, Wilmington, USA). First-strand complementary deoxyribonucleic acid (cDNA) was synthesized from 2 µg of total RNA using the NZY first-strand cDNA synthesis kit. Quantitative real-time PCR analysis was performed using a Xpert SYBR Green MasterMix (GRISP, Porto, Portugal) on a CFX Connect Real-Time PCR Detection System (Bio-Rad Laboratories, Inc). Gene expression was normalized to the housekeeping genes glyceraldehyde 3-phosphate dehydrogenase (GADPH) and hypoxanthine-guanine phosphoribosyltransferase 1 (HPRT-1) ranked as stably expressed by the RefFinder algorithm (https://www.heartcure.com.au/reffinder/). The relative expression of target genes was calculated based on the 2^−∆∆Ct^ method, where ΔΔCt=(Ct _target_−Ct _housekeeping_)_Sample_−(Ct _target_−Ct _housekeeping_)_Control_. Primer sequences (Eurofins Genomics, Lisboa, Portugal) are listed in Table 2. A Primer-BLAST search was performed to evaluate primer specificity and self-complementarity values of already validated and published primer sequences.

**Table 2:** Primer sequences used in qRT-PCR

Gene	Forward (5’ – 3’)	Reverse (5’ – 3’)
CCL2	CAAGATGATCCCAATGAGTAG	TTGGTGACAAAAACTACAGC
CXCL2	GGGTTGACTTCAAGAACATC	CCTTGCCTTTGTTCAGTATC
CXCR2	CTACTGCAGGATTAAGTTTACC	GACGTATATTACAACCACAGC
FN1	CCTATAGGATTGGAGACACG	GTTGGTAAATAGCTGTTCGG
GAPDH	GCCTTCCGTGGTCCTACC	GCCTGCTTCACCACCTTC
HPRT1	GTTGAAGATATAATTGACACT	GGCATATCCAACAACAAA
IL-6	TTCCATCCAGTTGCCTTC	TTCTCATTTCCACGATTTCC
IL-1β	TCTATACCTGTCCTGTGTAATG	GCTTGTGCTCTGCTTGTG
PRG4	GATAATGCTATTCCAGGCAC	CATCCAGAAATAATGACCTCG
S100A8	ATACAAGGAAATCACCATGC	ATATTCTGCACAAACTGAGG
S100A9	CTTTAGCCTTGAGCAAGAAG	TCCTTCCTAGAGTATTGATGG
TGF-β	ATGGTGGACCGCAACAAC	TTGCTATATTTCTGGTAGAGTTCC
TNF-α	CAAGGGACTAGACAGGAG	TGCCTCTTCTGCCAGTTC

### Western blot

Total protein extracts were prepared using standard lysis buffer, separated by sodium dodecyl sulfate/polyacrylamide gel electrophoresis (SDS-PAGE) and transferred to PVDF membranes (Bio-Rad, Hercules, CA, USA). Membranes were blocked with 5% of bovine serum albumin in Tris-buffered saline-tween 20 (TBS-T) and were incubated with appropriate primary antibodies fibronectin (1:500; ab2413; Abcam, Cambridge, UK), collagen IV (1:500; ab19808; Abcam, Cambridge, UK), EFEMP1 (1:1000, ab256457, Abcam), ITG β1 (1:1000, D6S1W, Cell Signalling), ITGα6 (1:1000, ab181551, Abcam) and CD44/HCAM (1:200,sc-7297, Santa Cruz Biotechnology), overnight at 4 °C. Afterward, the membranes were washed in 0.1% TBS-Tween and incubated with the secondary antibody (1:10000) for 1 h at RT and revealed by chemiluminescence (Clarity ECL western blotting substrates, BioRad) using ImageQuant LAS 500 chemiluminescence CCD camera (GE Healthcare Bioscience AB). Images were analysed using ImageJ software (National Institutes of Health).

### Adhesion assays

The 96-well plates were coated with fibronectin or collagen at concentrations ranging from 1 to 10 µg/mL for 2 h. The 143B-luc^+^ cells were seeded onto the substrates and allowed to adhere for 10 min at 37 °C. To remove the non-adherent cells, the wells were washed 3 times with PBS containing 1 M CaCl_2_.H_2_O and MgCl_2_.6H_2_O, and then 100 µL of EMEM culture medium containing 0.3 mg/mL of D-Luciferin was added to each well. The plate was read in the IVIS optical imaging system, and the bioluminescent signal was quantified using Living Image Software 4.10 (Xenogen, Alameda, CA, USA).

To evaluate the 143B cell adhesion to the decellularized lung scaffold, the decellularized fragments were placed in the 96-well plates and the 143B cells were seeded on top. The cells were allowed to adhere for 10 min at 37 °C and the same protocol as described above was followed to quantify cell adhesion.

### Mass spectrometry

#### Protein extraction

Proteins were reduced and alkylated with 100 mM Tris pH 8.5, 1% sodium deoxycholate, 10 mM tris(2-carboxyethyl)phosphine (TCEP), and 40 mM chloroacetamide for 10 min at 95ºC at 1000 rpm (Thermomixer, Eppendorf). 100 µg of protein were processed for proteomic analysis following the solid-phase-enhanced sample-preparation (SP3) protocol as described by Hughes et al. [37]. Enzymatic digestion was performed with Trypsin/LysC (2 µg) overnight at 37ºC at 1000 rpm. The resulting peptides were cleaned-up and desalted with C18 micro columns and further quantified.

#### Proteomics data acquisition

Protein identification was performed by nanoLC-MS/MS using an Ultimate 3000 liquid chromatography system coupled to a Q-Exactive Hybrid Quadrupole-Orbitrap mass spectrometer (Thermo Scientific, Bremen, Germany). Peptides were loaded onto a trapping cartridge (Acclaim PepMap C18 100Å, 5 mm x 300 μm i.d., 160,454, Thermo Scientific) in a mobile phase of 2% ACN, 0.1% FA at 10 µL/min. After 3 min loading, the trap column was switched in-line to a 50 cm by 75 μm inner diameter EASY-Spray column (ES803, PepMap RSLC, C18, 2 μm, Thermo Scientific, Bremen, Germany) at 300 nL/min. Separation was achieved by mixing A: 0.1% FA, and B: 80% ACN, with the following gradient: 5 min (2.5% B to 10% B), 120 min (10% B to 30% B), 20 min (30% B to 50% B), 5 min (50% B to 99% B) and 10 min (hold 99% B). Subsequently, the column was equilibrated with 2.5% B for 17 min. Data acquisition was controlled by Xcalibur 4.0 and Tune 2.9 software (Thermo Scientific, Bremen, Germany).

The mass spectrometer was operated in data-dependent (dd) positive acquisition mode alternating between a full scan (m/z 380–1580) and subsequent HCD MS/MS of the 10 most intense peaks from the full scan (normalized collision energy of 27%). ESI spray voltage was 1.9 kV. Global settings: use lock masses best (m/z445.12003), lock mass injection Full MS, chrom. peak width (FWHM) 15s. Full scan settings: 70k resolution (m/z 200), AGC target 3e6, maximum injection time 120 ms. dd settings: minimum AGC target 8e3, intensity threshold 7.3e4, charge exclusion: unassigned, 1, 8, > 8, peptide match preferred, exclude isotopes on, dynamic exclusion 45s. MS2 settings: microscans 1, resolution 35k (m/z 200), AGC target 2e5, maximum injection time 110 ms, isolation window 2.0 m/z, isolation offset 0.0 m/z, spectrum data type profile.

### Data analysis

The raw data was processed using the Proteome Discoverer software (Thermo Scientific) and searched against the UniProt database for Homo sapiens Proteome. The Sequest HT search engine was used to identify tryptic peptides. The ion mass tolerance was 10 ppm for precursor ions and 0.02 Da for fragment ions. The maximum allowed for missing cleavage sites was set to 2. Cysteine carbamidomethylation was defined as a constant modification. Methionine oxidation and protein N-terminus acetylation were defined as variable modifications. Peptide confidence was set to high. The processing node Percolator was enabled with the following settings: maximum delta Cn 0.05; decoy database search target FDR 1%, validation based on q-value. Imputation of missing values was performed only when a peptide was detected in at least one of the replicates analyzed. Quantitative evaluation was performed by pairwise comparisons of all detected peptides and the median ratio was used for protein level comparison. Significance assessment was performed using the background-based ANOVA method implemented in Proteome Discoverer 2.2 and multiple comparison adjustment of the p-values was performed.

### Protein functional enrichment analysis

The protein functional enrichment analyses for Gene Ontology (GO) terms and Reactome pathways were performed using the Database for Annotation, Visualization and Integrated Discovery (DAVID) 6.8 bioinformatics tool (https://david.ncifcrf.gov/summary.jsp). The data analysis and visualization of differently expressed proteins were conducted in the R programming language (version 4.1.1) in the RStudio integrated development environment. The GO and pathways plots were achieved using the ggplot2 (version 3.3.5). A basic circle packing chart with parckcircles package (available at https://r-graph-gallery.com/305-basic-circle-packing-with-one-level.html) was used for GO – biological processes. The Venn diagrams were generated with the webtool available at bioinformatics.psb.ugent.be/webtools/Venn/.

### Enzyme-linked immunosorbent assay (ELISA)

Levels of EFEMP1 ELISA in mouse serum samples and in the SCR of wild-type, MG-63 cells, 143B cells and EFEMP1-knockdown 143B cells were determined using the Human EFEMP1 ELISA kit (Abcam, ab269552) according to the manufacturer’s instructions.

### Kaplan-Meier analysis

The Kaplan-Meier analysis was performed using the R2 database (R2: Genomics Analysis and Visualization Platform – http://r2platform.com) which contains genome-wide gene expression data of OS patient samples (dataset: Mixed OS (Mesenchymal) – Kuijjer – 127 – vst – ilmnhwg6v2). The chondroblastic, fibroblastic and osteoblastic OS patient samples (n = 73) or with metastatic disease (n = 37) in the database were divided into high and low EFEMP1 (ID: 2202) expressions based on scan cut-off.

### Statistical analysis

All data are expressed as mean ± standard error of the mean (SEM). Graphics and statistical analysis were performed using GraphPad Prism version 8.0.2 (GraphPad Software, San Diego, CA, USA). Independent variables were analysed by the Mann-Whitney test, whereas Kruskal-Wallis or one-way ANOVA was used for multiple comparisons. Statistical significance was set at the level of p < 0.05. For the Kaplan-Meier analysis of OS patient samples, statistical differences in survival curves were calculated by log-rank (Mantel-Cox) test. Figure illustrations were created with adobe illustrator or BioRender.com.

## Results

### Osteosarcoma cell-derived secretome induces transcriptome and tissue structural changes similar to a primary growing tumour

**Fig. 1:**
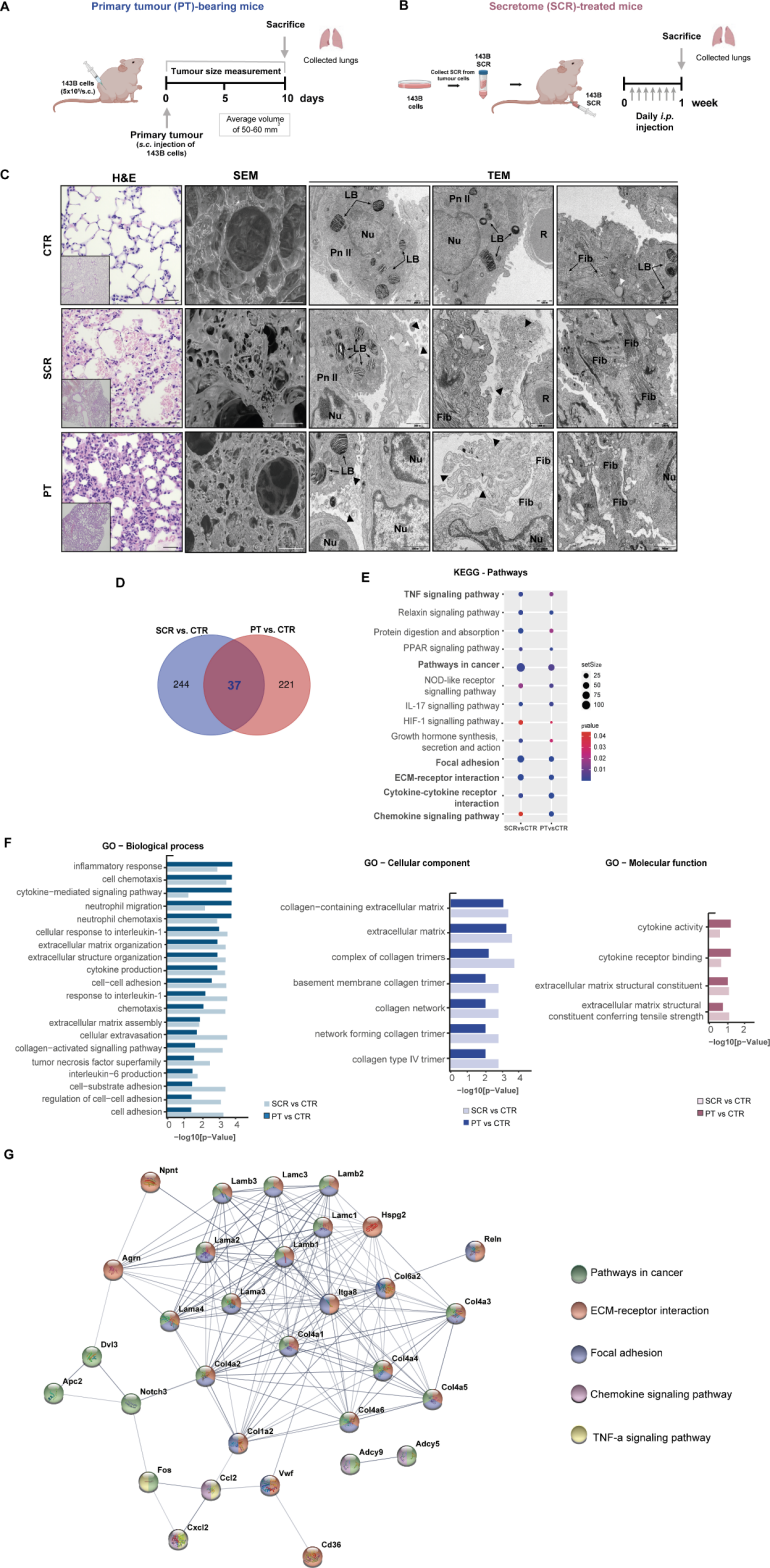
Changes in the transcriptome and lung tissue architecture in mice treated with the secretome or with a primary tumour. **A** Schematic diagram illustrating the *s.c.* injection of the 143B Luc^+^ cells into the lower flank of mice. Lung tissue was harvested when the primary tumour (PT) reached a maximum volume of 50–60 mm^3^ (PT-bearing mice, n = 3–5). **B** Schematic diagram illustrating the preparation and administration schedule of the 143B cell-derived secretome (SCR) in mice. Animals received daily *i.p.* injections for 1 week (SCR-treated mice, n = 3–5). Lung tissue was harvested at the end of the treatment. **C** Representative images of H&E at x200 magnification (Scale bar: 20 μm), SEM (Scale bar: 40 μm), and TEM (Scale bars: 1000 and 2000 nm) of lung tissue sections from untreated mice (CTR), SCR-treated or PT-bearing mice. Black arrowheads: exudate of protein, vesicles, and fragments of the surfactant; White arrowheads: mucous granules (produced by peribronchial glands); LB, lamellar bodies; Nu, nucleus; R, red blood cells; Pn II: pneumocytes type II (alveolar cells); Fib: fibrosis. **D** Venn diagram of differentially expressed genes (DEGs) in lungs for each pairwise comparison: SCR vs. CTR and PT vs. CTR. **E** KEGG pathway enrichment analysis of DEGs. Circle sizes denote the number of genes included in a group and the colour indicates the p-value. **F** Bar plots depicting the manually curated common Gene Ontology (GO) terms found for the two comparison groups. Biological process (BP), cellular component (CC), and molecular function (MF) of altered genes reporting the intersections in lungs from SCR vs. CTR and PT vs. CTR. **G** Representative protein-protein interaction (PPI) network, constructed with the common DEGs, using the STRING database

As the metastatic microenvironment plays a pivotal role in facilitating the seeding and outgrowth of disseminated tumour cells, we aimed to examine the lung environmental alterations that occur before lung metastasis formation. To establish the role of tumour-derived secreted factors in this process, we used two experimental mouse models. In the first group, animals were implanted subcutaneously with 143B OS cells for primary tumour (PT) formation (hereafter referred to as PT-bearing mice) (Fig. 1A). Tumours were allowed to grow until reaching an average volume of 50–60 mm^3^, which took approximately 8–10 days (Fig. S1A). To reproduce the continuous secretion of factors by developing primary tumour, a separate group of animals received daily *i.p.* injections of the secretome (SCR) derived from 143B cells (hereafter referred to as SCR-treated mice) for one week (Fig. 1B). In both conditions, none of the animals showed signs of respiratory distress or weight loss (Fig. S2A-B).

The lungs were subsequently harvested for histological and electron microscopic examinations. Histopathological analysis of formalin-fixed paraffin-embedded (FFPE) lung tissue from PT-bearing and SCR-treated mice demonstrated severe damage to the alveolar structure, with significant septum thickening compared to vehicle-treated control (CTR: 7.92 ± 0.40 μm; SCR: 15.79 ± 1.13 μm; PT: 24.25 ± 2.01 μm, p < 0.001), reduced airspace areas, and inflammatory cell infiltration, in comparison to the preserved lung parenchyma from control mice (Fig. 1C). No microscopically detectable lung metastasis was found in the lungs of PT-bearing mice at the endpoint.

Scanning electron microscopy (SEM) images revealed marked changes in the overall alveolar architecture with condensation of the connective tissue framework surrounding the alveolar spaces in the lungs of both PT-bearing and SCR-treated mice. Furthermore, transmission electron microscopy (TEM) images confirmed these observations and showed a disorganized and severely damaged pulmonary structure with leakage of proteins, vesicles, and surfactant (arrowheads) into the alveolar space. Additionally, septum thickening, nucleus with altered morphology, and fibrosis, evidenced by the accumulation of ECM proteins, in comparison with healthy controls.

These findings revealed early onset structural changes in the lung parenchyma, likely induced by the continuous release of distant tumour-secreted factors. Furthermore, the effects resulting from the daily administration of SCR closely resemble those observed in the host with the tumour, indicating a systemic tumour-mediated effect.

To understand the transcriptome alterations underlying lung histological and architectural changes, we performed comparative RNA sequencing-based transcriptome profiling of the lung tissue collected from healthy, SCR-treated, and PT-carrying mice.

Among the 16,138 transcripts, we found a total of 502 differentially expressed genes (DEGs) between the control and experimental groups, using a cut-off of p-value < 0.05 and log fold change (FC)>|1|. Of these, 37 were shared between the two compared groups as shown in the Venn diagram (Fig. 1D). This relatively limited overlap between the two groups may be due to the fact that the SCR was collected from cell monoculture and administered daily in constant volumes. Despite this approach mimicking the systemic release of secreted factors equivalent to a growing primary tumour during the PMN formation, might not fully replicate the secretion dynamics of a growing tumour or the influence of the surrounding stromal microenvironment on its composition.

Even though gene set enrichment analysis (GSEA) identified enriched pathways in cancer, ECM-receptor interaction, focal adhesion, chemokine signalling pathway, and cytokine-cytokine receptor interactions (Fig. 1E). Furthermore, gene ontology (GO) functional enrichment analysis of the common DEGs confirmed their significant contribution to several biological processes (BP) terms, including inflammatory response, cell chemotaxis, cytokine-mediated signalling pathway, neutrophil migration, ECM organization, cytokine production, cell-cell adhesion, cellular extravasation, interleukin-6 production, and cell adhesion (Fig. 1F). The common DEGs were also enriched in cellular components (CC) terms, specifically ECM and collagen, as well as in molecular functions (MF) terms, including ECM structural constituents, cytokine receptor binding and cytokine activity.

Furthermore, a protein-protein interaction (PPI) network analysis of the overlapping 37 DEGs identified 159 edges among 33 nodes (PPI enrichment p-value < 1.0 × 10^− 16^, using the String platform), with genes such as collagens (Col1, Col4 and Col6), laminins and integrin-alpha-8 as a part of the main cluster of ECM-receptor interactions, focal adhesion and pathways in cancer (Fig. 1G). Collagen, proteoglycans (e.g., versican and hyaluronan) and glycoproteins (e.g., laminins, elastin and fibronectin) are the core matrisome proteins of the ECM that maintain the proper tissue architecture [38]. Changes in ECM remodelling have profound implications for cellular signalling networks, as ECM components can act as pro-inflammatory stimuli and serve as ligands for various molecules and cell surface receptors, such as integrins, likely impacting metastasis formation [38–40]. The transcriptomic data strongly support this hypothesis, as several ECM-related pathways are dysregulated in response to secreted factors from the growing tumour or the administered secretome.

### Osteosarcoma-secreted factors cause changes in the lung ECM and the immune and inflammatory landscape during PMN formation

**Fig. 2:**
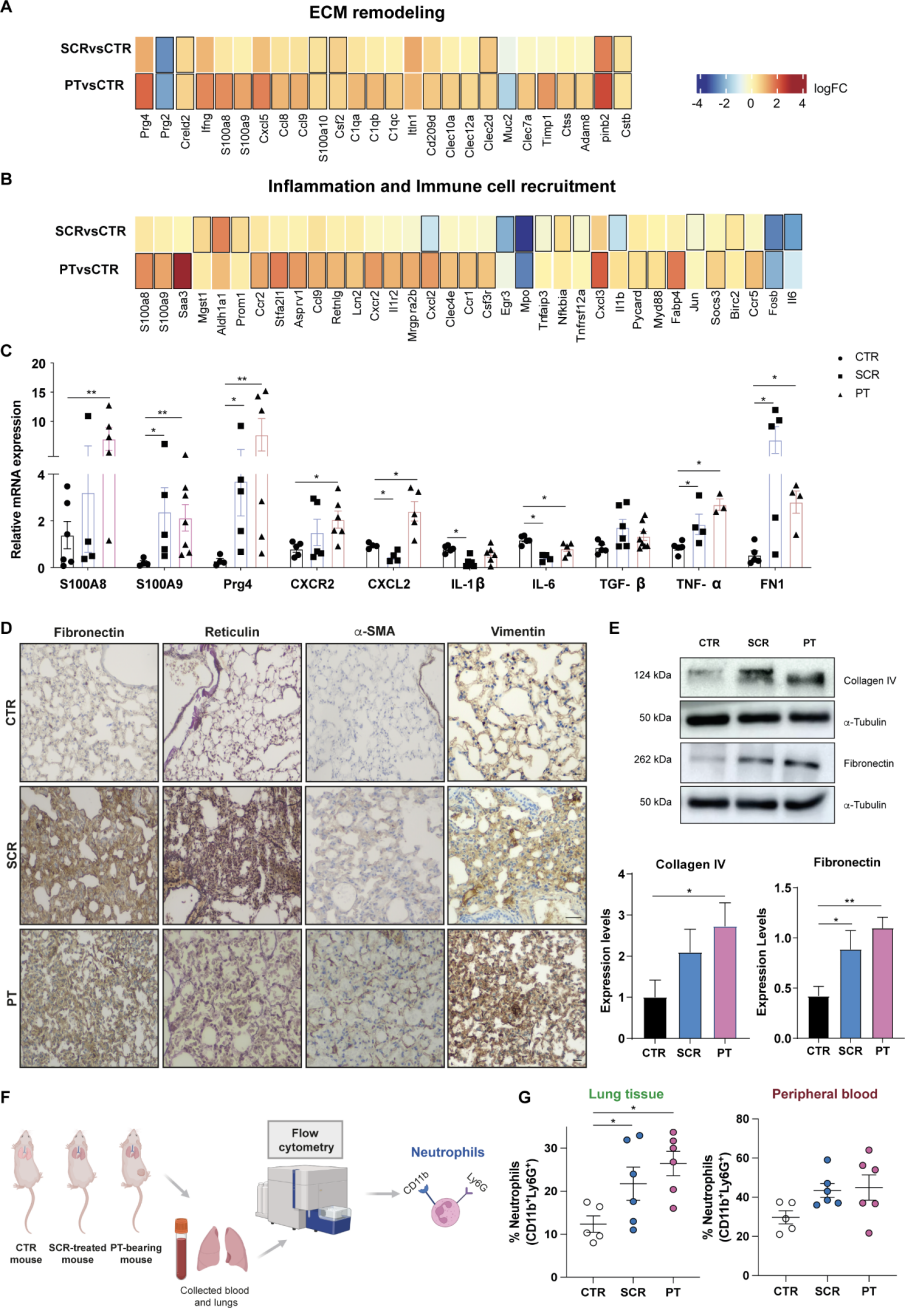
Lung microenvironmental changes in response to osteosarcoma-secreted factors. **A, B** Heatmaps of transcripts encoding genes involved in ECM remodelling, inflammation, and immune cell recruitment in lungs from SCR-treated or PT-bearing mice compared with controls. **C** qRT-PCR analysis of S100A8/A9, Prg4, Cxcr2, Cxcl2, IL-1β, IL6, TGF-β, TNF-α, and FN1 genes in lungs from SCR-treated mice or PT-bearing mice compared with controls. (n = 3–7, per group). **D** Representative images of fibronectin, reticulin, α-SMA, and vimentin immunostaining at x200 magnification (Scale bar: 30 μm) in lung sections from CTR mice, SCR-treated mice, or carrying a PT. **E** Western blot analysis of fibronectin and collagen type IV. Expression levels with graphic quantification. **F** Schematic diagram illustrating the analysis of lung-infiltrating neutrophils and the peripheral blood in mouse models by flow cytometry. **G** Flow cytometric quantification of infiltrating neutrophils in the lungs and in the peripheral blood of control, SCR-treated and PT-bearing mice (n = 5–6, per group). Data are presented as mean ± SEM from 3–7 independent biological samples. *p < 0.05, **p < 0.01 compared to control lungs (Mann-Whitney test)

After identifying the core biological pathways through GSEA, we performed a heatmap-based analysis of the relative expression of transcripts linked to ECM remodelling, inflammation, and immune cell recruitment-related pathways between the control and experimental groups. Transcripts with significant expression changes (p-value < 0.05) compared to control mice are marked with a black border. Lung transcriptomic analysis revealed a higher number of significantly dysregulated genes in mice with PT than in those treated with the SCR, although the general trend in gene dysregulation was quite similar (Fig. 2A-B). The heatmap of ECM-related genes revealed 26 deregulated genes (Fig. 2A). Notable findings include the upregulation of proteoglycan 4 (Prg4), known for its anti-inflammatory properties [41, 42], the downregulation of proteoglycan 2 (Prg2), a recognized tumour-suppressor [43], and the upregulation of cysteine-rich with EGF-like domain protein 2 (Creld2), which has implications for tumour progression via fibroblast reprogramming [44]. Other deregulated genes affiliated with the ECM and secreted ECM regulators include C1q complement components of collagen-like structures and C-type lectin-like receptors, the calcium-binding proteins S100A8/A9, the chemokines Cxcl5, Ccl8 and Ccl9, and the colony-stimulating factor (Csf) 2, all of them implicated in promoting cell proliferation, invasion and inflammation [45–49, 51, 52]. Within the heatmap of genes related to inflammation and immune cell recruitment, we found 35 deregulated genes (Fig. 2B), including chemokines, chemokine receptors and also the S100A8/A9, which are involved in the recruitment and activation of myeloid immune cells [50]. Myeloperoxidase (Mpo), an indicator of neutrophil activity [53], was downregulated, as was early growth response 3 (Egr3), a gene with tumour suppressor functions [53, 54]. Upregulated transcripts microsomal glutathione S-transferase (Mgst1) and aldehyde dehydrogenase 1 (Aldh1a1), together with lipocalin-2 (Lcn2), have been implicated in the immunosuppressive function of myeloid-derived suppressor cells (MDSCs) to facilitate tumour progression [55–58], while the fatty acid-binding protein 4 (Fabp4) has been strongly associated with cancer metastasis [49, 59].

Several of the differentially expressed transcripts were confirmed by qRT-PCR in lung tissue lysates, including S100A8/A9, Prg4, Cxcr2, Cxcl2, IL-1β, and IL6, and the results were consistent with RNA-seq data (Fig. 2C). Additionally, we examined the mRNA expression of TGF-β, TNF-α, and fibronectin, known for their immunosuppressive, pro-angiogenic, and ECM regulatory roles, respectively, which are also upregulated in the lungs of PT-bearing and SCR-treated mice compared to the controls.

Immunohistochemical (IHC) analysis of lung tissue sections revealed a marked increase in the deposition of two major ECM components, fibronectin and reticulin fibers, as well as upregulation of the activated fibroblast marker α-SMA and vimentin in both SCR-treated and PT-bearing mice, compared to the control group (Fig. 2D). It was also observed an increase in type IV collagen, (the major component of the basement membrane) by western blot (WB), as well as of fibronectin (Fig. 2E). These findings support the transcriptomic evidence of lung ECM remodelling during PMN formation.

After identifying DEGs enriched in biological processes related to the recruitment of myeloid cells, we conducted a flow cytometry analysis of infiltrating neutrophils and other immune cells (macrophages, eosinophils, monocytes and B-lymphocytes) in the lungs and peripheral blood of control, SCR-treated, and PT-bearing mice (Fig. 2F). The results showed a significant increase in the percentage of infiltrating neutrophils (CD11b^+^ Ly6G^+^) in the lungs of PT-bearing or SCR-treated mice compared to age-matched untreated mice (Fig. 2G). A similar trend was observed in the peripheral blood, albeit without reaching statistical differences. Regarding other immune cell populations (monocytes, macrophages, eosinophils and B-lymphocytes), no significant differences were observed in either the lungs or peripheral blood among the different experimental groups (Fig. S3A-B).

Depending on the microenvironment, neutrophils can acquire an anti-tumorigenic N1 or a pro-tumorigenic N2 phenotype [60]. In our study, we could not identify the phenotype of lung-infiltrating neutrophils, but the increased mRNA levels of TGF-β, a major regulator of neutrophil polarization, suggest an N2-like phenotype. Moreover, the transcriptomic analysis revealed an upregulation of Lcn2, a secreted glycoprotein produced by N2-neutrophils and implicated in promoting metastasis [61, 62], simultaneously with a marked downregulation of Mpo in the premetastatic lungs, providing additional support for this hypothesis.

Altogether, these findings suggest that tumour-secreted factors instigate inflammatory signalling pathways in the lung microenvironment with concurrent ECM remodelling and neutrophil infiltration as part of PMN formation.

### Activated lung fibroblasts in premetastatic lungs drive fibronectin and collagen deposition and favour the adhesion of OS cells to the lung ECM

Since fibroblasts are the predominant cells in the lung interstitium and the major producers of ECM components, we hypothesized that tumour-secreted factors trigger the activation of stromal fibroblasts for ECM remodelling.

**Fig. 3:**
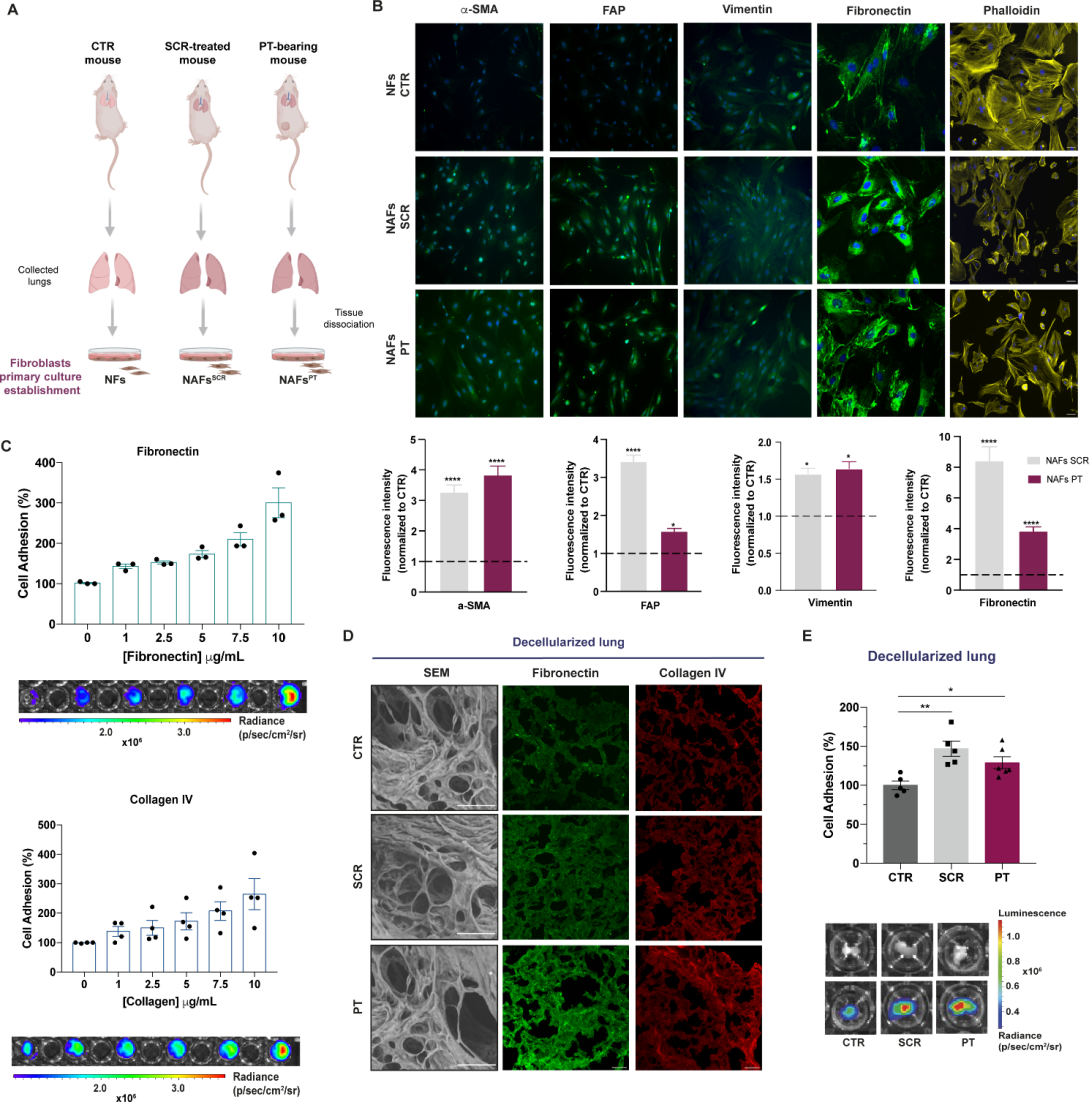
Increased deposition of fibronectin and collagen by activated lung fibroblasts favour the adhesion of OS cells to the lung ECM. **A** Schematic diagram of the establishment of the primary cultures of fibroblasts isolated from the lungs of untreated (NFs), SCR-treated (NAFs^SCR^) and PT-bearing mice (NAFs^PT^). **B** Representative image of immunofluorescence staining for α-SMA, FAP, vimentin, fibronectin and phalloidin at x20 magnification (Scale bar: 50 μm) in primary cultures of fibroblasts from CTR, SCR-treated or PT-bearing mice. Immunofluorescence quantification of α-SMA, FAP, vimentin and fibronectin (n = 3, per group). **C** Relative adhesion of 143B cells to increasing concentrations of fibronectin and collagen type IV ranging from 1 to 10 µg/mL (n = 3–4, performed in triplicate). Representative bioluminescence images of cell adhesion (143B cells) to different concentrations of fibronectin and collagen IV. **D** Representative SEM images (Scale bar: 50 μm) of decellularized lung sections and fibronectin and collagen immunostaining at x20 magnification (Scale bar: 50 μm) in decellularized lung sections from CTR mice, SCR-treated mice, or carrying a PT. **E** Relative adhesion of 143B cells to decellularized lung sections from CTR, SCR-treated, or PT-bearing mice (n = 5). Representative pictures of the decellularized fragments in the wells (upper row) and bioluminescent images of adhered cells (lower row). The bioluminescent signal is represented as radiance (p/s/cm^2^/sr). Data are presented as mean ± SEM. *p < 0.05 and ****p < 0.0001 are significantly different when compared to NFs (Kruskal-Wallis (B)); *p < 0.05 and **p < 0.01 when compared to healthy decellularized lungs (One-way ANOVA (E))

To address this, we performed a phenotypic profiling analysis of lung fibroblasts directly isolated from freshly excised lungs of SCR-treated and PT-bearing mice, as schemed in Fig. 3A. Fibroblasts from age-matched healthy mice were used as controls and were termed as normal fibroblasts (NFs). Immunostaining showed an upregulation of α-SMA and FAP, two classical markers of activated fibroblasts, as well as of the intermediate filament vimentin, in fibroblasts from SCR-treated animals or PT-bearing animals, hereafter referred to as normal activated fibroblasts, NAFs^SCR^ or NAFs^PT^, respectively. The strong immunoreactivity for fibronectin in NAFs^SCR^ and NAFs^PT^, further supports the contribution of stromal fibroblasts to lung ECM remodelling, as previously described. Furthermore, staining with phalloidin, a marker for F-actin filaments, revealed alterations in actin organization, characterized by an irregular and disorganized shape and shortening of filament structures (Fig. 3B) in fibroblasts from PT-bearing and SCR-treated animals. These phenotypic changes were accompanied by an increase in cell motility, as assessed by a wound healing assay (Fig. S4A-B).

Given that fibronectin is a cell-adhesive glycoprotein known to promote tumour cell adhesion, we hypothesized that secretome-induced upregulation of fibronectin might promote further lung colonization by OS cells. To address this, we performed a cell adhesion assay with 143B cells on fibronectin-coated plates using coating concentrations ranging from 1 to 10 µg/mL. The results showed a gradual increase in the percentage of adherent cells with increasing fibronectin concentrations. Similar results were observed on collagen IV-coated plates, underscoring the role of these proteins as substrates for cell attachment (Fig. 3C).

Encouraged by these findings, we evaluated the adhesion of 143B cells to decellularized lung fragments from SCR-treated or PT-bearing mice. Immunofluorescence staining confirmed the increased deposition of fibronectin and collagen IV in decellularized fragments of animals treated with the SCR or with a PT (Fig. 3D), in agreement with IHC and WB data (Fig. 2D, E). We then seeded 143B cells on plates coated with decellularized lung scaffolds and allowed them to adhere for 10 min. Our results showed a significant increase in the percentage of 143B cells attached to the lung scaffolds of both SCR-treated (p < 0.01) and PT-bearing mice (p < 0.05), representing an approximately 50% increase compared to control animals (Fig. 3E). Furthermore, immunostaining of decellularized fibroblast cell sheets revealed an increase in staining intensity for fibronectin and collagen IV (Fig. S5), confirming the contribution of activated fibroblasts to ECM remodelling and cell adhesion.

### Tumour secretome-induced microenvironmental changes in pre-metastatic lungs promote and accelerate lung metastasis formation

**Fig. 4:**
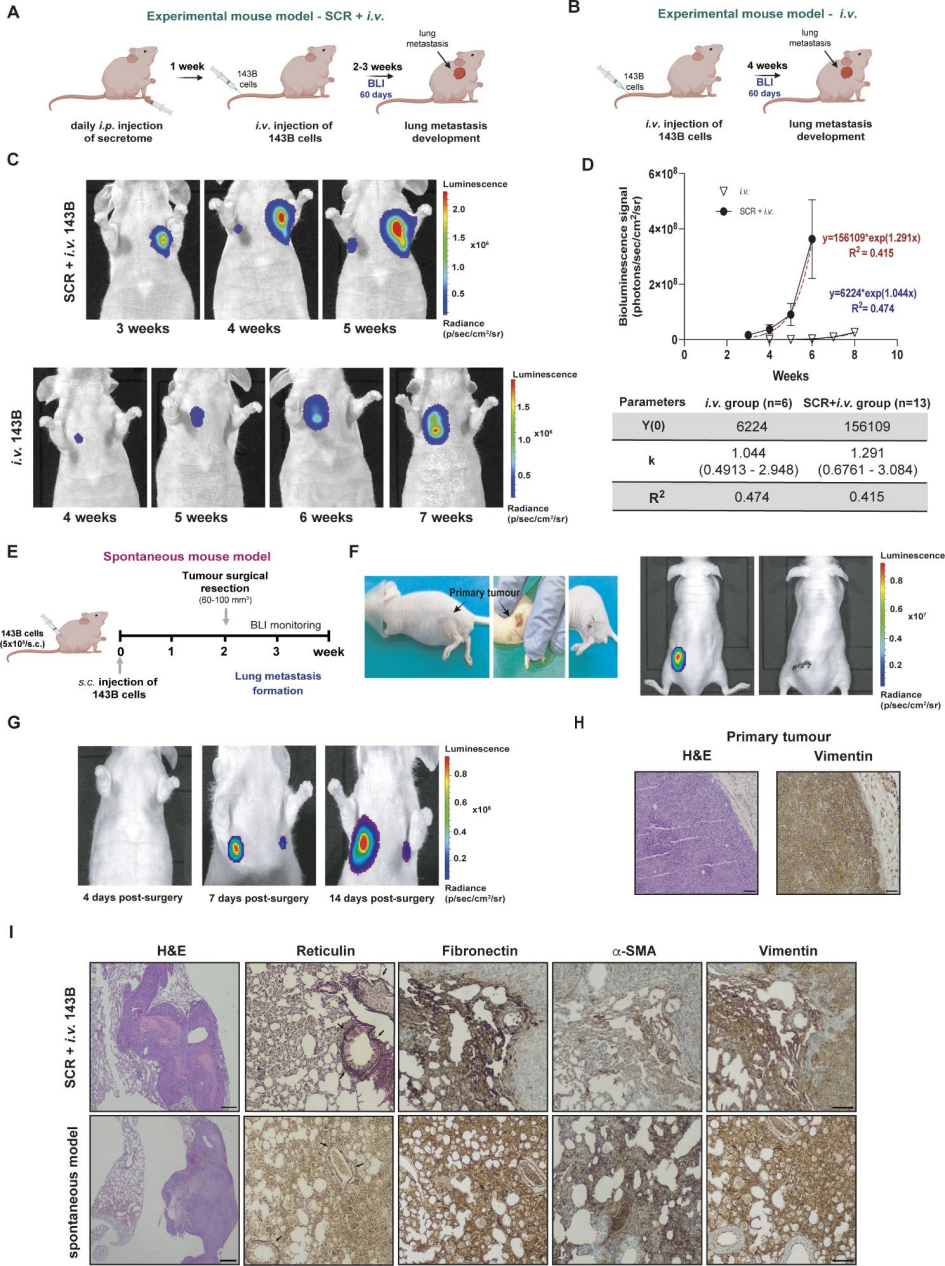
Osteosarcoma-induced PMN formation promotes and accelerates the formation of lung metastasis. **A** Schematic diagram of the experimental model of lung metastasis. Mice were treated with 143B cells-derived secretome (SCR) for 1 week, followed by *i.v.* administration of 143B Luc^+^ cells into the tail vein (SCR + *i.v.* group). **B** Schematic diagram of the experimental model of lung metastasis. Mice received only the *i.v.* injection of 143B Luc + cells without pre-treatment with the SCR (*i.v.* group). **C** Representative bioluminescence images of lung metastasis formation in pre-treated (SCR + *i.v. group)* and untreated (*i.v.* group) mice with secretome before the *i.v.* injection of 143B cells. **D** Exponential fitting of the bioluminescence signals (photons/second) of metastatic lesions over time in the *i.v.* group (∇ n = 6) and the SCR + *i.v.* group (• n = 13 mice), and corresponding kinetic parameters. **E** Schematic diagram of the spontaneous metastatic mouse model. Animals were injected subcutaneously with the 143B cells. After reaching a volume of 60–100 mm^3^, the tumour was excised, and the animals were monitored by BLI for lung metastasis formation. **F** Images of the surgical resection of a primary tumour with a volume of 60 mm^3^ and bioluminescence images before and after the excision of the tumour. **G** Representative bioluminescence images at 4, 7 and 14 days after surgical resection of the primary tumour. **H** Histological H&E images at x100 magnification (Scale bar: 30 μm) and immunostaining for vimentin at x100 magnification (Scale bar: 30 μm) of the resected tumour. **I** Histological H&E images at x40 magnification (Scale bar: 20 μm) and IHC staining for reticulin (arrowed), fibronectin, α-SMA, and vimentin at x100 magnification (Scale bar: 30 μm) of lung metastatic lesions in both experimental and spontaneous mouse models. Data are presented as mean ± SEM

Up to this point, our data showed the impact of the OS cell secretome on lung PMN formation. To confirm whether this effect promotes and/or accelerates lung metastasis formation, we used an experimental metastasis model in mice. Animals were treated with the 143B-derived secretome daily for one week, followed by the *i.v.* injection of tumour cells into the tail vein (Fig. 4A). Non-preconditioned mice were used as the controls (Fig. 4B). Lung metastasis formation was monitored weekly for over 60 days using bioluminescence imaging (BLI).

All preconditioned animals developed lung metastasis, detected in the second to third weeks after cell inoculation, which rapidly progressed to larger and multilobular lesions. In contrast, only 55% of the untreated group, developed metastasis by the fourth week, mostly unilobular, with slower growth kinetics, never reaching the size of the treated group (Fig. 4C). Parameters estimated from the exponential fitting of the bioluminescent signals confirmed a faster growth rate of metastatic lesions in animals pre-treated with the OS cells secretome (Fig. 4D). The development of metastasis in some untreated animals was probably due to the highly aggressive nature of the143B cells. However, the metastatic rate was substantially lower and the lesions were smaller and less invasive than those in the preconditioned group.

We also established a spontaneous metastatic animal model to assess whether PMN formation induced by an engrafted primary tumour dictate the innate propensity of OS to metastasize to the lung. A primary tumour was induced by subcutaneous injection of 143B cells into the lower flank and allowed to grow until it reached a maximum volume of 60–100 mm^3^ (Fig. 4E). The tumour was then surgically excised and the animals were monitored for lung metastasis formation. Post-operative BLI images confirmed the complete removal of the primary tumour (Fig. 4F) and the absence of lung metastasis or metastasis to other organs. Four days post-surgery, there were no signs of lung metastasis; however, a week later, two nodules were detected, which progressively evolved over the following two weeks (Fig. 4G). These results suggest that, at the time of surgery, tumour cells were already disseminated in the circulation at the time of surgery and were able to colonize the permissive lung microenvironment induced by the primary tumour, allowing for their survival and metastatic outgrowth. The spontaneous formation of distant lung metastasis further confirms the organotropism of OS in this organ, which is thought to be achieved by factors secreted by the engrafted OS cells.

Histopathological examination confirmed the high-grade malignancy in both primary OS (Fig. 4H), and lung metastasis in both the experimental and spontaneous models (Fig. 4I), as evidenced by marked nuclear pleomorphism, the high mitotic rate, and intralesional necrotic areas. The Gordon-Sweet staining of lung sections, using silver impregnation, revealed an increased deposition and rearrangement of fibrillar collagens surrounding the metastatic lesions and around the bronchi and blood vessels, consistent with a fibrotic response.

Additionally, IHC showed strong immunoreactivity for fibronectin and α-SMA in the stromal regions of the periphery of the neoplastic lesions, which revealed the presence of activated fibroblasts in the lung. The mesenchymal marker vimentin, also known as fibroblast-intermediate filament, exhibited strong immunoreactivity in stromal and metastatic cells, confirming the mesenchymal origin of the latter.

Overall, these findings confirmed that lung alterations induced by the secretome or grafted OS cells create a favourable environment for the survival and outgrowth of metastatic cells and undergo continuous remodelling.

### Analysis of the proteomic profile of cell secretome and mouse plasma identified EFEMP1 as a potential prognostic biomarker in osteosarcoma

Since the effects of daily administration of SCR on a premetastatic lung were quite similar to those elicited by a local primary tumour, we performed a comparative mass spectrometry-based label-free quantitative proteomic analysis of the 143B SCR and the plasma from PT-bearing mice to identify potential common mediators predictive of lung metastasis formation. Secretome collected from a non-metastatic OS cell line (MG-63) [63] and plasma from healthy mice were used as controls. We confirmed the non-metastatic potential of MG-63 cells in mice, as none of the animals *i.v.* injected with these cells developed metastases (Fig. S6A).

A list of differentially expressed proteins (DEPs) of 143B *versus* MG-63 OS cells and plasma from healthy mice *versus* PT-bearing mice was generated using an FDR-adjusted p-value ≤ 0.05. A total of 2,595 proteins were identified in the secretome of OS cells, with 109 proteins differentially expressed between the 143B and MG-63. In the mouse plasma, we considered only proteins of human origin to focus on what was secreted by human OS-induced tumours. Out of these, we identified 195 differentially expressed between the plasma samples of PT-bearing mice and healthy controls.

**Fig. 5:**
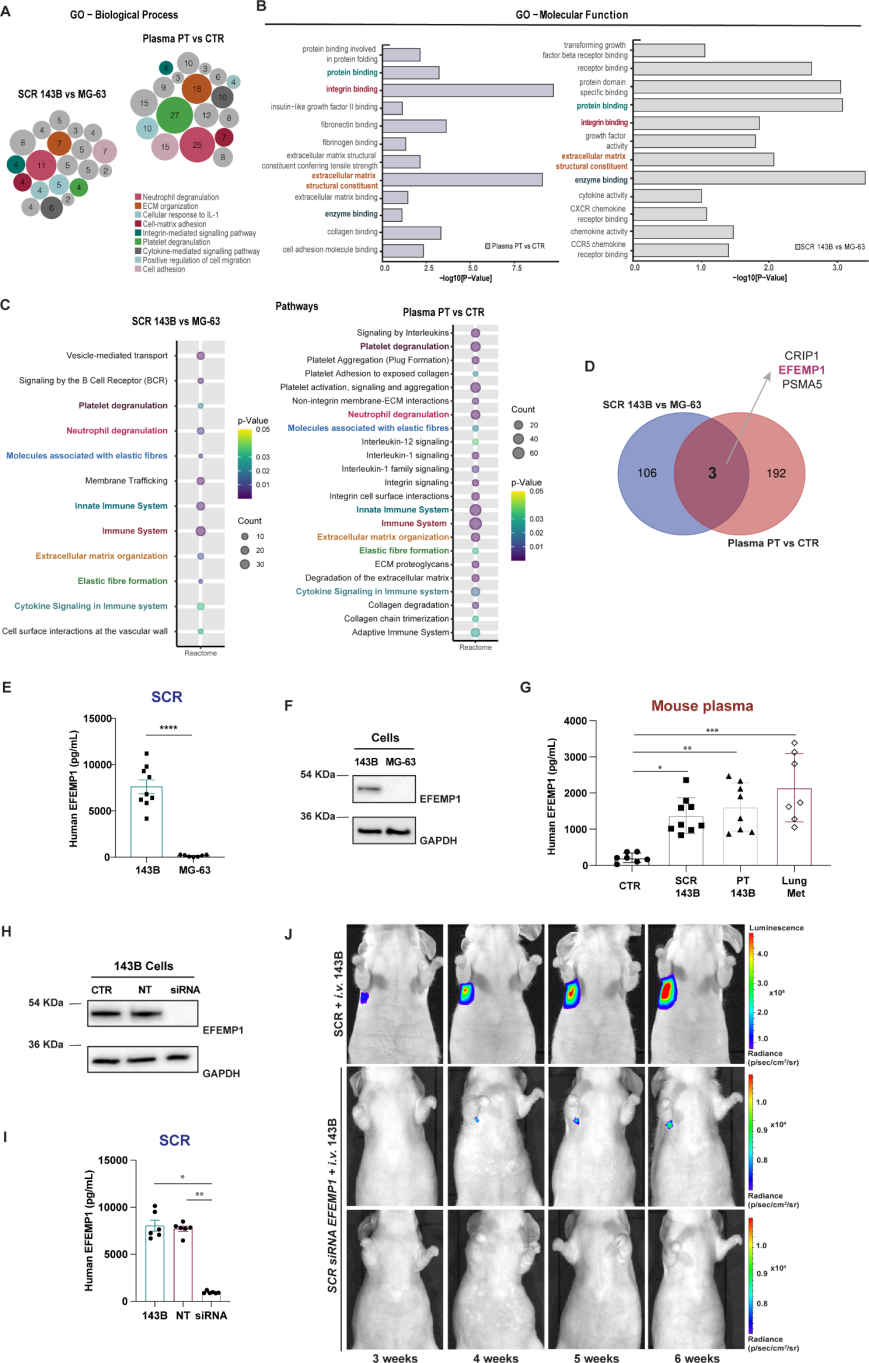
Proteomic analysis identified EFEMP1 as a potential metastatic-related biomarker in osteosarcoma. **A, B** Gene ontology analysis (GO) output. Biological process (BP) and molecular function (MF) of differently expressed proteins (DEPs) in the two pairwise-comparison groups (SCR 143B vs. MG-63; Plasma PT vs. CTR). Circle sizes in BP denote the number of genes involved in the process. **C** Reactome pathway enrichment analysis of identified proteins in the two pairwise- comparison groups. Circle sizes denote the number of genes included in a group and colour indicates the p-value. The common pathways are highlighted. **D** Venn diagram showing specific and common proteins among the two pairwise groups: SCR 143B vs. MG-63 and Plasma PT vs. CTR. **E** EFEMP1 levels in the SCR of the metastatic 143B and non-metastatic MG-63 OS cells. **F** Representative western blot of EFEMP1 in the metastatic 143B and non-metastatic MG-63 OS cells. **G** Plasma levels of EFEMP1 in control mice (CTR), mice treated with the 143B SCR, bearing a primary tumour (PT) or with lung metastasis (Lung Met). **H** Representative western blot of EFEMP1 expression in 143B cells (CTR), non-targeting (NT) and siRNA knockdown of EFEMP1 cells. **I** EFEMP1 levels in the SCR of 143B cells, NT and siRNA EFEMP1-knockdown cells. **J** Representative bioluminescence images of mice treated with the SCR of control 143B (SCR + *i.v. group)* or EFEMP1-knockdown cells (SCR siRNA EFEMP1 + *i.v.* group) followed *i.v.* injection of 143B-Luc^+^ cells. Data are presented as mean ± SEM, from 7 to 8 independent experiments. **** p < 0.0001 were significantly different when compared with the SCR from MG-63 (unpaired t-test (E)); *p < 0.05, **p < 0.01 and ***p < 0.001 were significantly different when compared with the values present in the plasma from healthy mice (Kruskal-Wallis test (G); *p < 0.05, **p < 0.01 were significantly different when compared with 143B SCR (Kruskal-Wallis test (I))

GO enrichment analysis was performed on DEPs between the two pairwise comparisons to identify shared biological processes and molecular functions. The largest number of identified proteins was particularly enriched in neutrophil degranulation, platelet degranulation, ECM organization, and cell adhesion (Fig. 5A-B). Reactome enrichment analysis identified 8 common overlapping pathways engaged in platelet degranulation, neutrophil degranulation, molecules associated with elastic fibres, innate immune system, immune system, ECM organization, elastic fibre formation and cytokine signalling in the immune system (Fig. 5C). All of these pathways are known to be involved in PMN formation [64–67]. The Venn diagram in Fig. 5D identified three overlapping DEPs between the two pairwise comparisons, which include the epidermal growth factor-containing fibulin-like extracellular matrix protein 1 (EFEMP1, also called fibulin-3), cysteine-rich protein 1 (CRIP1) and proteasome subunit alpha type-5 (PSMA 5). Among these proteins, EFEMP1 was the most abundant in both the SCR and mouse plasma, exhibiting consistently high-intensity peak values and greater protein coverage across all replicates. EFEMP1 is an ECM glycoprotein broadly expressed in various tissues during development and adulthood. As a crucial component of the basement membrane, EFEMP1 plays a crucial role in preserving the structural integrity and stability of the ECM [68]. Considering the ECM alterations in the lungs of mice challenged with the SCR or carrying a PT, we reasoned that secreted EFEMP1 could play an important role in this process. To address this, we validated our proteomic approach by measuring protein expression and secreted levels of EFEMP1 by OS cells *in vitro.*

ELISA and western blot analysis confirmed the exclusive expression and secretion of EFEMP1 by 143B cells, with negligible levels observed in the non-metastatic MG-63 cell line, thereby supporting the proteomic data (Fig. 5E, F). Moreover, EFEMP1 was detected in the plasma of animals treated with the secretome or carrying a PT, as well as in those with established lung metastasis, with higher levels observed in the latter (Fig. 5G). The use of a human EFEMP1 ELISA kit points to 143B cells as a source of the plasmatic EFEMP1 levels. Importantly, this protein is involved in 3 out of the 10 common biological processes, specifically ECM organization, molecules associated with elastic fibres and elastic fibre formation, suggesting that it may contribute to ECM remodelling during metastasis.

To evaluate the functional effects of secreted EFEMP1 in lung metastasis formation, we knocked down 143 cells for EFEMP1 and collected the SCR. The siRNA knockdown resulted in effective protein silencing and a robust 90% reduction in EFEMP1 secretion, as confirmed by Western blot and ELISA, respectively (Fig. 5H, I). Non-targeting siRNA did not affect EFEMP1 expression or secretion. A new group of animals was then preconditioned with the SCR of EFEMP1-silenced cells and then injected into the tail vein with 143B-Luc ^+^ cells, as previously described. Remarkably, this approach resulted to the complete prevention of lung metastasis formation in 80% (4/5) of the animals throughout the 40-day monitoring period by BLI. The single case of metastasis that did occur was observed at later time-points and exhibited a slower progression rate compared those received the whole-secretome (Fig. 5J).These data demonstrate that EFEMP1 is indeed required to foster a permissive PMN to support the colonization, survival, and proliferation of incoming cells.

**Fig. 6:**
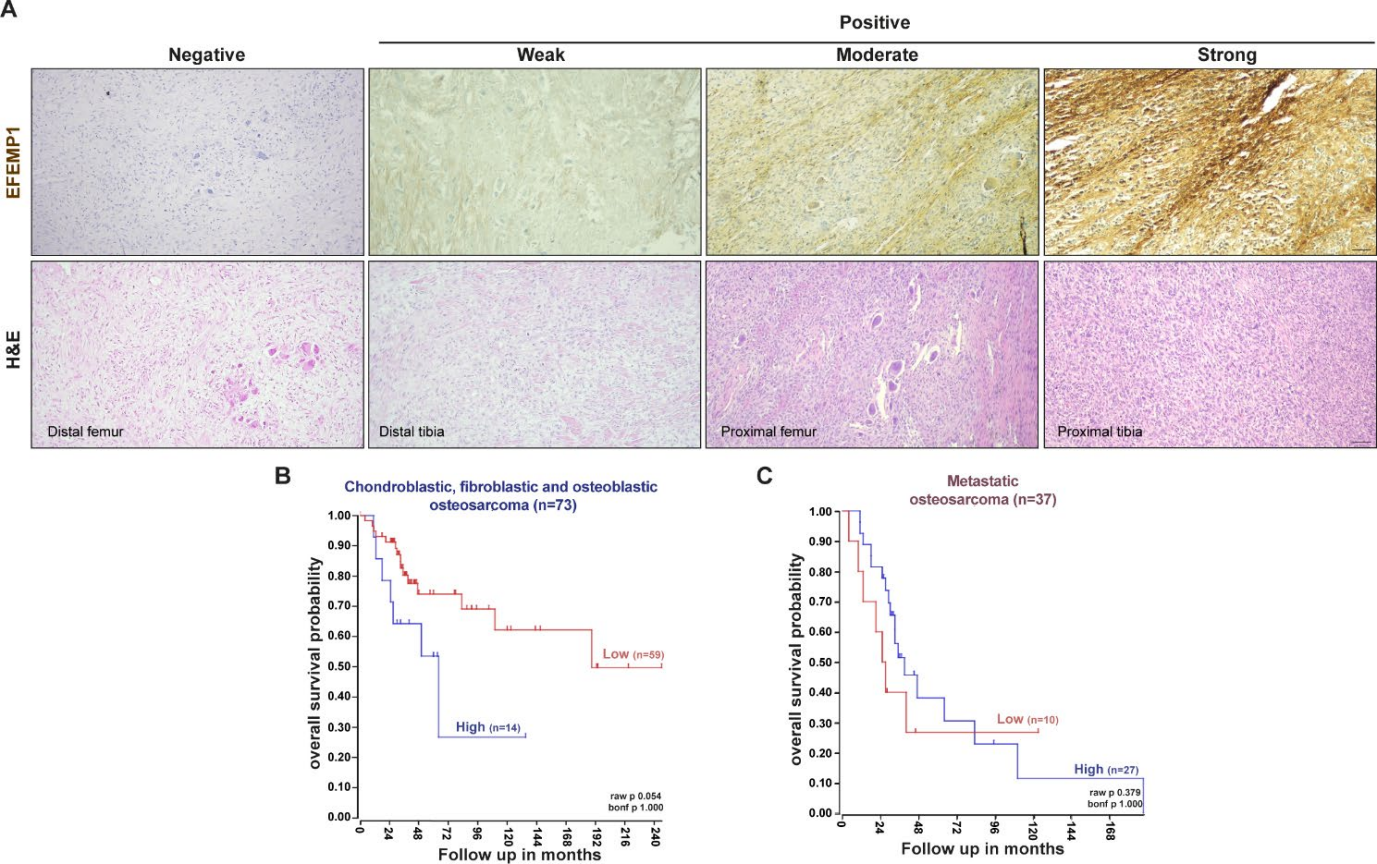
EFEMP1 expression in primary tumours correlates with poor prognosis in OS patients. **A** Representative images of EFEMP1 staining and H&E in biopsy samples of high-grade OS patients at x100 magnification (Scale bar: 50 μm). **B, C** Kaplan-Meier analysis of overall survival in chondroblastic, fibroblastic and osteoblastic OS patient samples (n = 73 patients) and with metastatic disease (n = 37 patients). Scan cut-off was used to group samples into high (blue) and low (red) EFEMP1 expressions. p-values were determined by a log-rank test

To explore the potential prognostic significance of EFEMP1 expression in OS patients, we conducted a retrospective study on a small cohort of 29 patients without evidence of metastasis at the time of diagnosis. Archived paraffin-embedded biopsy specimens of the primary tumour were immunohistochemically stained for EFEMP1 to investigate its association with the occurrence of lung metastasis during the follow-up period. Of the 29 patients, 12 (41.4%) experienced lung metastasis, and 50% of these patients had positive biopsy specimens for EFEMP1 with a disease-free interval (DFI) of 38.5 months (range: 6–77 months). The other 6 patients who tested negative for EFEMP1 had a longer DFI of 44 months (range: 15–159 months). Among the 17 patients who did not develop lung metastasis, only 6 (35%) showed positive staining for EFEMP1. Importantly, patients with EFEMP1-positive biopsies had a higher mortality rate of 58% compared to those with negative biopsies, whose mortality rate was 29%. Representative images of H&E and immunostaining for EFEMP1 in biopsy samples with negative, weak, moderate and strong intensities are shown in Fig. 6A. Positive staining was primarily observed in the cytoplasm and membrane of OS cells.

Due to the small sample size in our study, we were unable to perform a reliable Kaplan-Meier survival analysis. Therefore, we used the recognized R2 bioinformatic platform to conduct a univariate analysis, which revealed a significant correlation between high EFEMP1 expression and poorer overall survival (246 vs. 135 months) in high-grade conventional OS patients (raw p-value = 0.05, Fig. 6B). However, in the case of patients with lung metastases, we did not find a significant correlation between EFEMP1 expression and the overall survival (raw p-value = 0.379, Fig. 6C). These findings suggest that EFEMP1 upregulation in primary tumours is associated with poor prognosis in OS patients, particularly those with high-grade conventional OS. However, further studies using larger sample sizes are required to confirm these results and understand the underlying mechanisms of EFEMP1 in cancer progression and patient outcomes.

## Discussion

In this study, we aimed to understand the mechanisms by which OS cells reprogram the lung microenvironment to support colonization and outgrowth of disseminated tumour cells. Employing two experimental mouse models and a multi-omics approach, we demonstrated that OS cell-derived secreted factors instigate a permissive lung PMN for incoming tumour cells, comprising ECM remodelling, fibroblasts activation, neutrophil recruitment and shaping of the inflammatory environment. These alterations, observed in animals carrying a primary tumour or treated with SCR, highlight the pivotal role of OS-secreted factors in setting up the lung metastatic process.

The transcriptome data identified several DEGs enriched in related biological processes, indicating ECM remodelling, inflammatory response, cell adhesion and chemotactic signals as essential steps. IHC analysis confirmed the increased deposition of fibronectin and fibrillar collagen in the lungs of both experimental mouse models, a result that was further verified in decellularized lung fragments. These proteins, in addition to forming the ECM backbone, act as anchor points for cell adhesion molecules, like β1 and α6 integrins, as well as CD44, all of which are expressed in 143B OS cells (Fig. S7). Indeed, the increased attachment of 143B cells to decellularized lung scaffolds, confirms the pivotal role of fibronectin and collagen in mediating cell adhesion, regarded as a critical step for organ colonization (Fig. 3C, E). The remodelling of the ECM is also implicated in metastatic organotropism through organ-specific integrin-ECM interactions. Exosomal α6β4 and α6β1 integrins have been shown to dictate the exosome adhesion to ECM components and lung resident fibroblasts, resulting in stromal remodelling to host metastatic cells [69]. Although, we have not explored the individual contribution of exosomes to PMN formation, recent studies from our group confirmed that they express β1 and α6 integrins as their parental cells and have an intrinsic homing ability for homotypic lung metastasis [27].

Fibroblasts are the main producers of ECM components, both in homeostasis and in response to injury. In tumours, they are the major players in dysregulated collagen turnover and fibronectin deposition, manifested as desmoplasia [70–72]. Our findings confirmed a fibroblast-to-myofibroblast transition during the PMN, with concomitant deposition of fibronectin and collagen, that persists throughout metastatic progression. It is worth noting that these fibroblasts were isolated directly from tumour-free lung tissue without any intervening culture steps that might have impacted gene expression, further confirming the ability of fibroblasts to functionally adapt to the evolving environment. Cancer-associated fibroblasts (CAFs) are the most prominent stromal cells and serve as positive regulators of tumour growth at the primary tumour site by reprogramming the local microenvironment [73–75]. Similarly, metastasis-associated fibroblasts (MAFs) have been found to promote metastatic outgrowth by engaging in inflammatory-mediated communication with cancer cells [76, 77]. These findings were obtained in models with established lung macrometastases, whereas in our study, the fibroblasts phenotypic changes were observed in an incredibly early stage before tumour cell colonization.

The cytokine TGF-β is a major inducer of myofibroblast differentiation through autocrine and paracrine mechanisms, resulting in the secretion of additional growth factors, cytokines and chemokines involved in a complex integration of signals promoting the recruitment of BMDCs, angiogenesis, immunosuppression, and ECM remodelling [78, 79]. Transcriptome and RT-PCR analyses confirmed the expression of TGF-β and other fibroblast activation inducers (e.g. TNF-α and CCl2) in premetastatic lungs, suggesting the fibroblast contribution in sustaining an inflammatory response. These findings are consistent with a study by Mazumdar et al. [80] on highly metastatic OS cell lines, including 143B, where they found that OS-derived EVs induced lung myofibroblast differentiation through the activation of the TGF-β1 and SMAD2 pathways. Flow cytometric analysis showed a significant increase in the infiltration of CD11b^+^Ly6G^+^ neutrophil (but not of other immune cells) in the pre-metastatic lungs of both models compared with age-matched controls. Similar findings were reported by Hiratsuka et al., who showed that TNF-α-induced S100A8 and A9 signalling promotes the expression of serum amyloid A3 (SAA3) and the recruitment of CD11b^+^ myeloid cells to premetastatic lungs [50, 81]. A large number of clinical studies have correlated high levels of intratumoral and systemic neutrophils with poorer disease prognosis, attributed to the release of pro-angiogenic and tumour-growth factors and the suppression of immune responses [82–84]. A few studies have reported the accumulation of neutrophils in future metastatic sites, but their role at this early stage remains elusive as both pro- and anti-metastatic functions have been described [26, 85–87]. As part of the innate immune system, neutrophils are recruited to sites of inflammation driven by cytokines and chemotactic factors to modulate the inflammatory process. Transcriptomic and qPCR analyses identified expression of several members of the CXC and CC chemokines and S100A8/A9 that are chemotactic for neutrophils, which could explain their accumulation in premetastatic lungs, but not their functional significance. Nevertheless, the upregulation of TGF-β and the marked downregulation of neutrophil MPO suggest a pro-tumorigenic role rather than anti-metastatic protection. A limitation of this study is the use of immunocompromised mice, which does not allow us to assess the contribution of neutrophils to the suppression of T cell-mediated responses. However, inhibition of Ly6G + cell mobilization has been shown to prevent lung metastasis formation in both immunocompetent and immunodeficient mice, suggesting a T cell-independent effect, at least in the early stages [86]. However, further studies are needed to clarify the pro-metastatic contribution of neutrophils to PMN formation.

The follow-up animal studies confirmed that systemic remodelling of the lung microenvironment in the SCR-treated and PT-bearing mice promoted and accelerated lung metastasis formation, suggesting that a pre-existing growth-permissive niche is crucial for the incoming of metastatic cells.

Comparative analysis of differentially secreted proteins in the SCR of 143B cells and plasma samples of PT-bearing mice, identified common significantly enriched GO terms related to ECM organization, cell-matrix adhesion, neutrophil and platelet degranulation, and cytokine-mediated signalling. These findings are consistent with lung transcriptomic GO terms and, more importantly, with the lung microenvironmental changes previously described.

The glycoprotein EFEMP1 was one of the enriched proteins that was found in the 143B cell SCR, and in the plasma of mice with primary OS, lung metastasis or treated with the 143B cell-derived SCR in a tumour-free setting, suggesting a driving oncogenic role for lung metastasis in a premetastatic stage. The suppression of lung metastasis formation observed in 80% (4/5) of mice preconditioned with EFEMP1-depleted SCR, strongly supports this hypothesis, and importantly confirm the crucial role of this secreted protein in providing a permissive microenvironment, which is required for the subsequent survival and outgrowth of OS cells in the lung.

As already mentioned, EFEMP1 plays a central role in maintaining the structural integrity of the ECM, organizing the ECM scaffold and in promoting cell-ECM adhesion in normal connective tissue and disease [88]. This protein is upregulated in some tumours and has been described as a novel soluble signal strongly associated with the malignant progression in gliomas, pancreatic, ovarian, and bladder cancer, as well as OS, by promoting cell proliferation, MMP-induced invasion, migration, EMT, and angiogenesis [89–93]. Although not explored, we reasoned that EFEMP1 is involved in ECM remodelling and/or contributes to cell-matrix adhesion, which are integral parts of the premetastatic process, as previously observed in our study. Moreover, EFEMP1 is part of the core effectors of these biological processes. These findings point to EFEMP1 as a putative therapeutic target and a novel plasma biomarker with added value in predicting the risk of lung metastasis in OS. However, further mechanistic studies are warranted to understand the significance of EFEMP1-dependent SCR in favouring the metastatic potential of OS cells and to dissect the interplay between tumour and stromal cells mediated by EFEMP1.

In clinical studies, the small size of our patient cohort precluded us from establishing a definite causal relationship between EFEMP1 expression in primary tumour and the development of lung metastases. Nevertheless, we observed that patients with EFEMP1-expressing tumours have poorer overall survival, suggesting an association with tumour aggressiveness, which is substantiated by the Kaplan-Meier survival analysis. Further research with larger patient cohorts is needed to confirm this hypothesis and gain a more comprehensive understanding of EFEMP1 involvement in the disease.

## Conclusions

In summary, our study sheds light on systemic-induced lung microenvironmental changes that precede metastatic spread in OS. Integration of our data uncovers neutrophil infiltration and the functional contribution of stromal-activated fibroblasts in ECM remodelling for tumour cell attachment as early pro-metastatic events, which may hold therapeutic potential in preventing or slowing metastatic spread. Moreover, we identified EFEMP1, a secreted protein by OS cells, as a potential driver of lung metastasis. It not only holds promise as a therapeutic target but also as a plasma biomarker with added value in predicting the risk of lung metastasis in OS.

### Electronic supplementary material

Below is the link to the electronic supplementary material.

Supplementary Material 1

## Data Availability

The datasets generated in this study are available in the following databases: RNA-Seq data: GEO database [GSE216744] (https://www.ncbi.nlm.nih.gov/geo/query/acc.cgi?acc=GSE216744). Protein identification: PRIDE database [PXD040814]. (https://www.ebi.ac.uk/pride/archive/projects/PXD040814)

## Abbreviations

BLI: Bioluminescence imaging
BMDCs: Bone marrow-derived cells
BP: Biological Process
CAFs: Cancer-associated fibroblasts
CC: Cellular components
DAVID: Database for Annotation, Visualization and Integrated Discovery
DEGs: Differently expressed genes
DEPs: Differentially expressed proteins
DFI: Disease-free interval
ECM: Extracellular matrix
ELISA: Enzyme-linked immunosorbent assay
EVs: Extracellular vesicles
FDR: False discovery rate
FFPE: Formalin-fixed paraffin-embedded
GO: Gene ontology
GSEA: Gene set enrichment analysis
*i.p.*: Intraperitoneal
*i.v*.: Intravenous
IHC: Immunohistochemical
KEGG: Kyoto Encyclopedia of Genes and Genomes
MAFs: Metastasis-associated fibroblasts
MF: Molecular Function
NAFs: Normal activated fibroblasts
NFs: Normal fibroblasts
OS: Osteosarcoma
PMN: Premetastatic niche
PPI: Protein-protein interaction
PT: Primary tumour
RT: Room Temperature
SCR: Secretome
SEM: Scanning electron microscopy
STRING: Search Tool for the Retrieval of Interacting Genes/Proteins
TEM: Transmission electron microscopy
WB: Western blot
