# Supplementary material for: Unveiling the role of osteosarcoma-derived secretome in premetastatic lung remodelling
Source: J Exp Clin Cancer Res. 2023 Nov 30;42:328. doi: 10.1186/s13046-023-02886-9 (PMC10688015; doi:10.1186/s13046-023-02886-9)
Supplement: Supplementary file 1 — Supplementary Material 1 [file 13046_2023_2886_MOESM1_ESM.docx]

**ADDITIONAL FILE 1**

**SUPPLEMENTARY FIGURES**


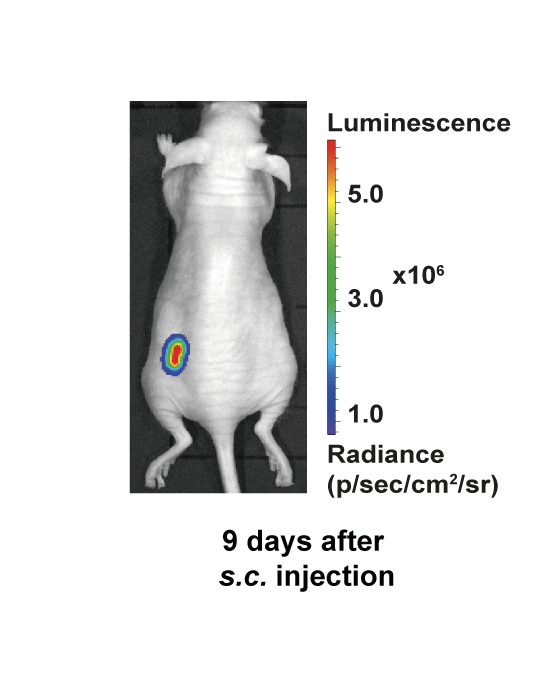


**Fig. S1. *In vivo* bioluminescence (BLI) imaging of subcutaneous primary tumour.** BLI monitoring of subcutaneous primary tumour formation 9 days after *s.c.* injection of 143B-luc^+^ cells. The bioluminescent signal is represented as radiance (photon/s/cm^2^/sr).

**
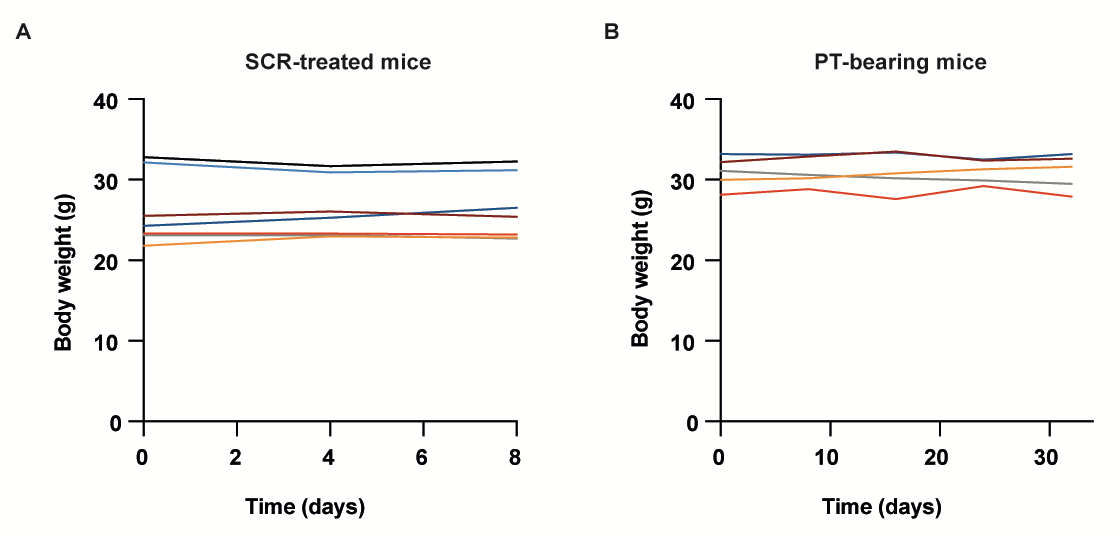
**

**Fig. S2. Body weight analysis in SCR-treated and PT-bearing mice. A** Monitoring of body weight over 8 days in the SCR-treated mice. **B** Monitoring of body weight over 30 days in the PT-bearing mice.

**
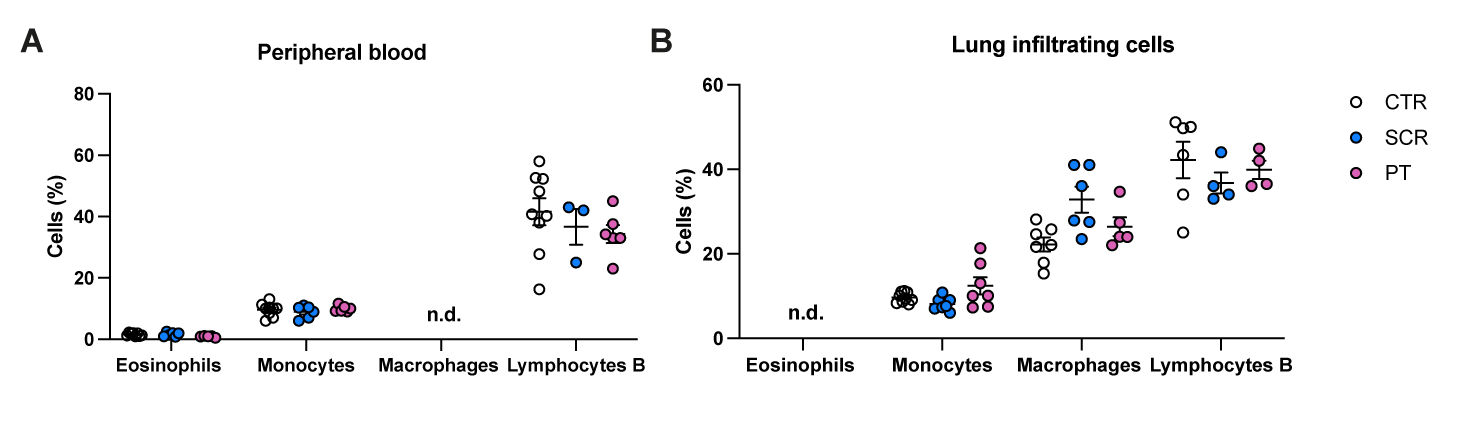
**

**Fig. S3.** Flow cytometric quantification of infiltrating immune cells in the peripheral blood **(A)** and in the lungs **(B)** of control, SCR-treated and PT-bearing mice (n=5-6, per group).

**
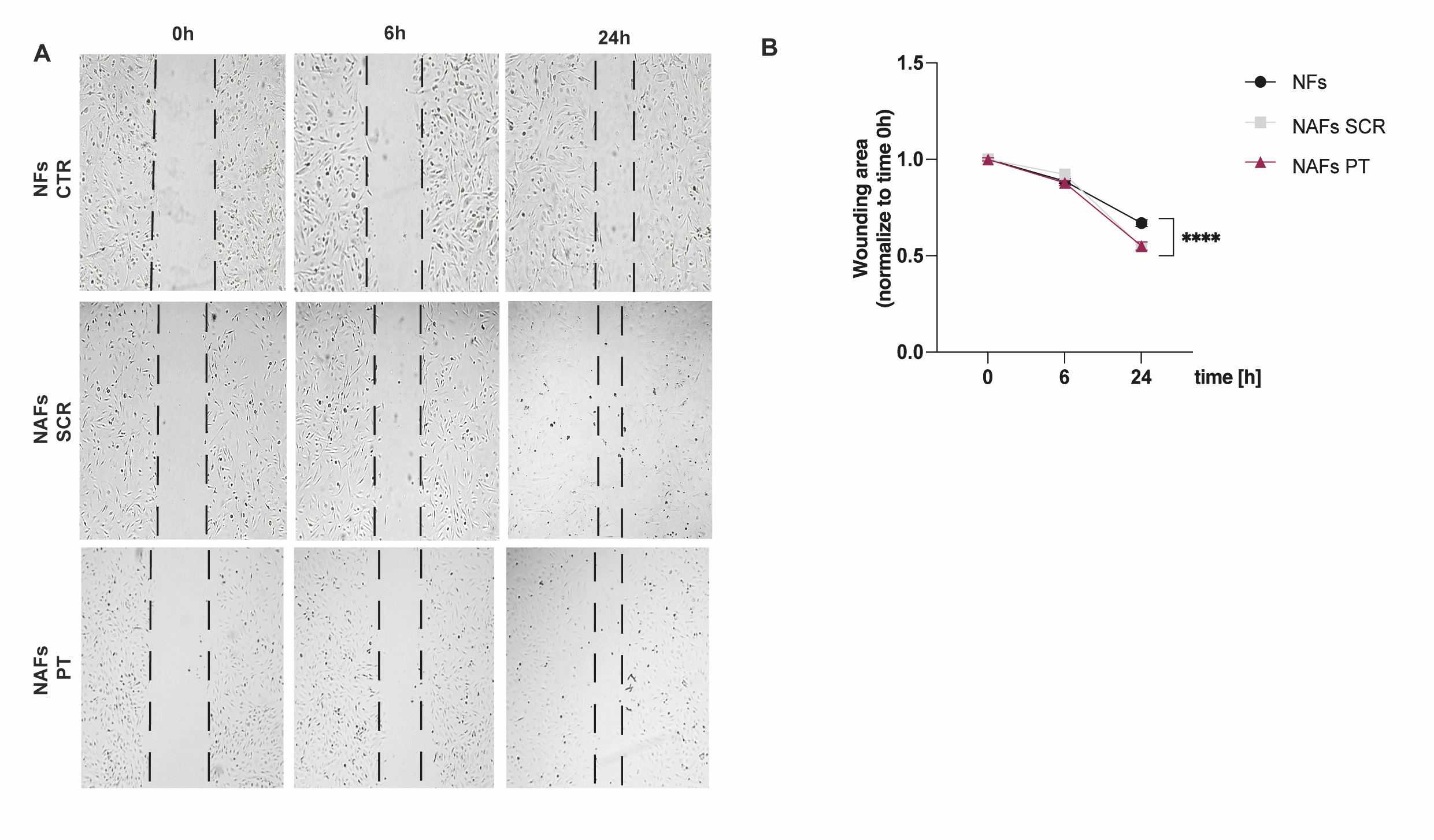
**

**Fig. S4. Cell migration (wound healing) assay in primary cultures of fibroblasts isolated from lungs of untreated (NFs), SCR-treated (NAFs^SCR^) and PT-bearing mice (NAFs^PT^). A** Representative images of scratch closure assay of lung fibroblasts monolayers at 0, 6 and 24 h. **B** Quantification of scratch closure assay expressed as the remaining area uncovered by the cells. The scratch is at time point 0 h was set to 1. ****p<0.0001 significantly different when compared with NFs using a non-parametric Kruskal-Wallis test (n=3).


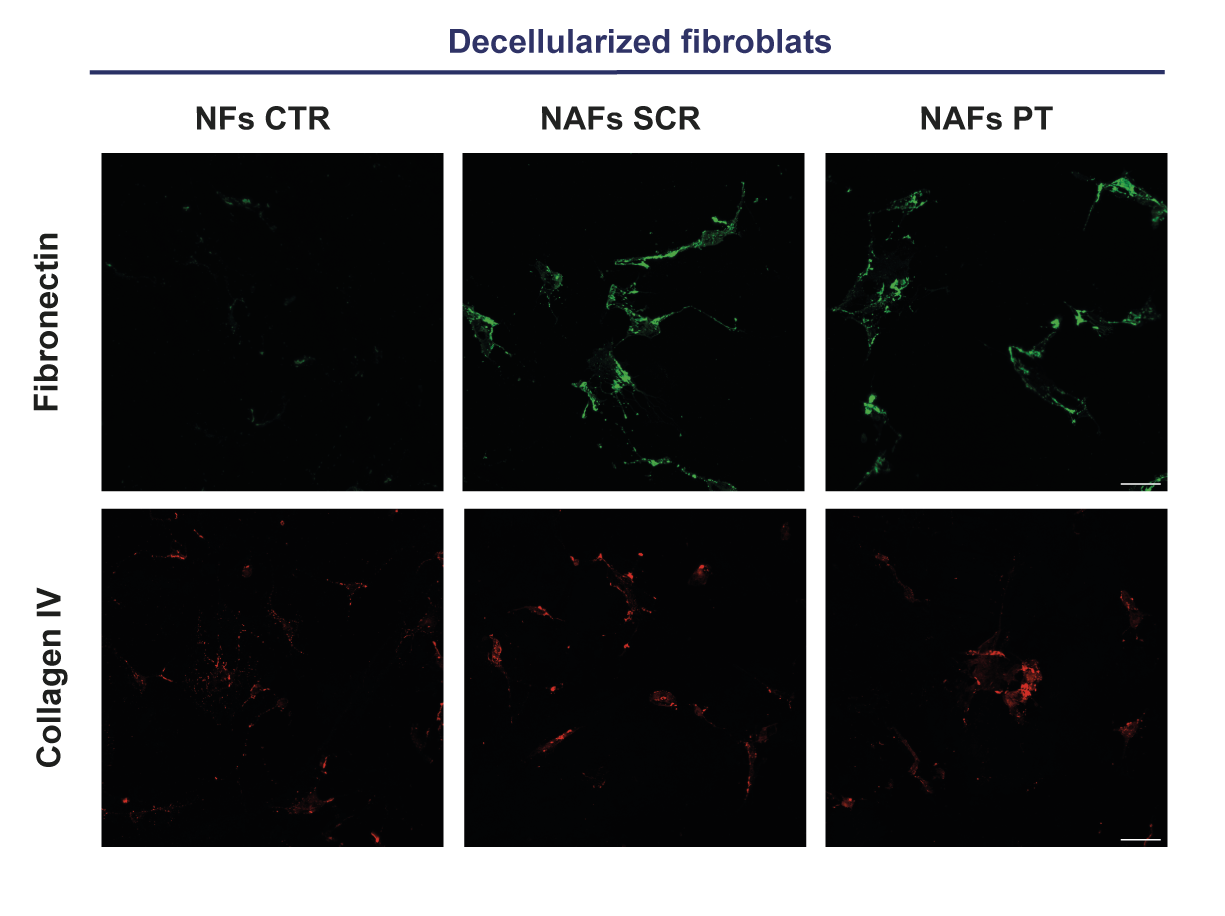


**Fig. S5. Fibronectin and collagen IV depositions in decellularized lung fibroblast cell sheets from SRC-treated, PT-bearing mice and CTR mice.** Representative immunofluorescence images of decellularized lung fibroblasts stained for fibronectin and collagen IV at x20 magnification (Scale bar: 50 µm) from CTR, SCR-treated or PT-bearing mice (n=3).


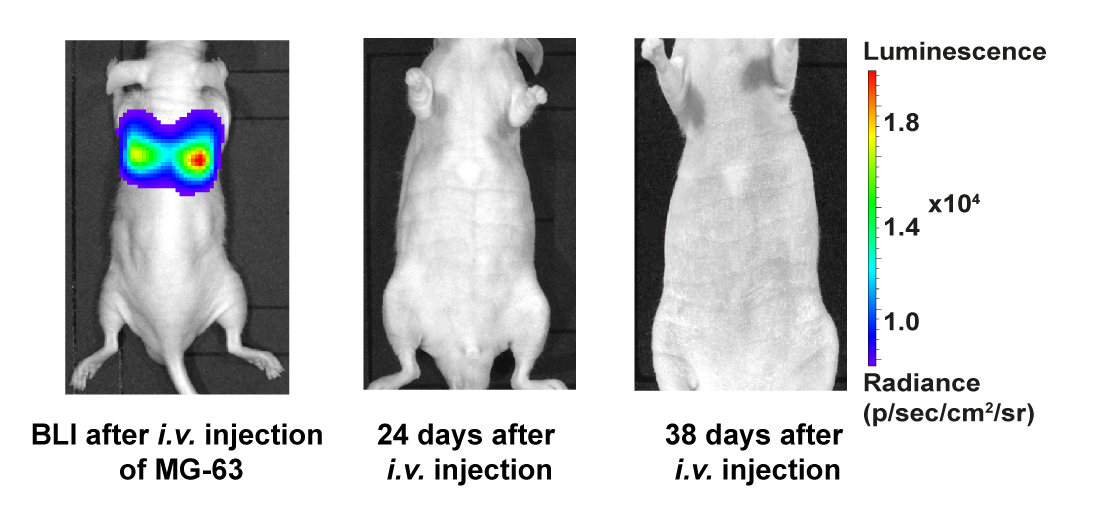


**Fig. S6. *In vivo longitudinal* bioluminescence (BLI) imaging of pre-treated mice with MG-63-derived secretome following *i.v.* injection of MG-63-luc^+^ cells (1.5 x 10^6^ cells).** BLI images acquired immediately after the *i.v.* injection of MG-63-luc^+^ cells showing the arrival of tumour cells to the lungs and BLI images at days 24 and 38 after *i.v.* injection of cells without evidence of lung metastasis formation. The bioluminescent signal is represented as radiance (photon/s/cm^2^/sr).


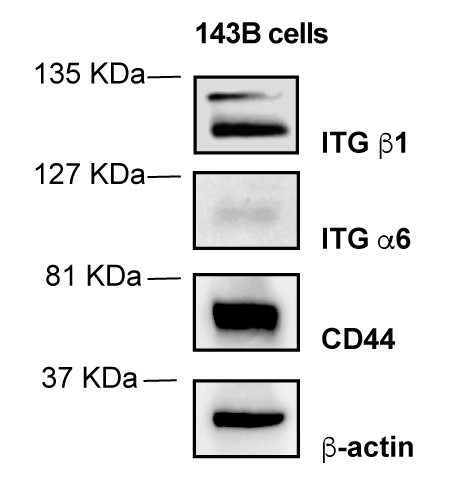


**Fig. S7. Western blot analysis of key cell adhesion proteins in 143B cells.** Representative western blot of ITG β1, ITG α6 and CD44 proteins in 143B cells.
